# New Estrone Oxime Derivatives: Synthesis, Cytotoxic Evaluation and Docking Studies

**DOI:** 10.3390/molecules26092687

**Published:** 2021-05-04

**Authors:** Catarina Canário, Mariana Matias, Vanessa Brito, Adriana O. Santos, Amílcar Falcão, Samuel Silvestre, Gilberto Alves

**Affiliations:** 1 CICS-UBI–Health Sciences Research Centre, University of Beira Interior, 6200-506 Covilhã, Portugal; catarina_canario@hotmail.com (C.C.); mariana.r.matias@gmail.com (M.M.); vanessa_12_479@hotmail.com (V.B.); aos@ubi.pt (A.O.S.); gilberto@fcsaude.ubi.pt (G.A.); 2 Laboratory of Pharmacology, Faculty of Pharmacy, University of Coimbra, 3000-548 Coimbra, Portugal; acfalcao@ff.uc.pt; 3 CIBIT-Coimbra Institute for Biomedical Imaging and Translational Research, University of Coimbra, 3000-548 Coimbra, Portugal; 4 CNC–Center for Neuroscience and Cell Biology, University of Coimbra, 3004-504 Coimbra, Portugal

**Keywords:** estrone, oximes, cytotoxicity, cancer, docking

## Abstract

The interest in the introduction of the oxime group in molecules aiming to improve their biological effects is increasing. This work aimed to develop new steroidal oximes of the estrane series with potential antitumor interest. For this, several oximes were synthesized by reaction of hydroxylamine with the 17-ketone of estrone derivatives. Then, their cytotoxicity was evaluated in six cell lines. An estrogenicity assay, a cell cycle distribution analysis and a fluorescence microscopy study with Hoechst 3358 staining were performed with the most promising compound. In addition, molecular docking studies against estrogen receptor α, steroid sulfatase, 17β-hydroxysteroid dehydrogenase type 1 and β-tubulin were also accomplished. The 2-nitroestrone oxime showed higher cytotoxicity than the parent compound on MCF-7 cancer cells. Furthermore, the oximes bearing halogen groups in A-ring evidenced selectivity for HepaRG cells. Remarkably, the Δ^9,11^-estrone oxime was the most cytotoxic and arrested LNCaP cells in the G_2_/M phase. Fluorescence microscopy studies showed the presence of condensed DNA typical of prophase and condensed and fragmented nuclei characteristic of apoptosis. However, this oxime promoted the proliferation of T47-D cells. Interestingly, molecular docking studies estimated a strong interaction between Δ^9,11^-estrone oxime and estrogen receptor α and β-tubulin, which may account for the described effects.

## 1. Introduction

Cancer is a major public health problem and is one of the leading causes of death worldwide [1]. Therefore, over the years, medicinal chemists and other researchers have been working in the development of new drugs with antitumor activity—namely, starting from molecules that already exist in nature [2]. In this context, steroids are natural compounds that are usually involved in cell proliferation and consequently in cancer development [3]. In the 90s, several steroids having very unusual and interesting structures were isolated from marine sponges. Among these, steroidal oximes isolated by Rodriguez et al. [4] from *Cinachyrella* sponges showed relevant antiproliferative activity against several types of cancer cells [5]. The interesting results observed in these studies stimulated researchers to prepare different series of steroidal oximes with potential anticancer interest. Classically, the oxime functional group is usually introduced by condensation of an aldehyde or a ketone, including of steroidal origin, with hydroxylamine affording, respectively, aldoximes and ketoximes [6].

Among the different groups of steroidal oximes developed as anticancer agents, several of them have endocrine activity, acting by inhibition of steroid sulfatase (ST), aromatase, 17α-hydroxylase-17,20-lyase (CYP450_17α_), 5α-reductase (5α-R) or 17β-hydroxysteroid dehydrogenase type 1 (17β-HSD1) enzymes [7,8,9,10,11,12]. ST converts estrone sulfate into estrone (E1) and therefore its inhibition can be of interest in breast cancer treatment. In this context, Hejaz and co-workers [8] synthesized the estrone oxime sulfamate (OMATE), which inhibited the ST enzyme and showed a potency similar to the observed with the ketone analogue estrone sulfamate (EMATE). Later, modified 2-substituted estrogen-3-*O*-sulfamate 17-oximes have also been prepared and showed significant antiproliferative activity against breast MCF-7 cells [13]. 17β-HSD type 1 enzyme reduces the 17-ketone of estrane steroids to the corresponding 17β-hydroxylated derivatives [14]. Relevant 17β-HSD1 inhibition was also demonstrated by Allan *et al.* for 16-oxime and 6,17-oximes of E1 (% inhibition = 96% and 83%, respectively, at 10 μM) [9,12]. Interestingly, docking studies performed by these authors showed that both the presence of 16 and 17-oximes were associated to an improved 17β-HSD1 inhibition [9]. Also, estrone (E1)-16-oxime ethers showed antiproliferative activities against cervix (HeLa), ovarian (A2780), MCF-7 and epidermoid (A431) cancer cell lines, promoted an apoptotic cell death and modulated the cell cycle progression (arrest at phase G_1_) [15]. Estrogen receptor (ER), which is a transcription factor, was also involved in cell proliferation. Wendlandt et al. [16] showed that oxime estrogen dimers are able to effectively enter cells and modulate ERα-mediated genomic signaling. Otherwise, 2-methoxyestradiol (2ME2) is a naturally occurring E2 derivative with antitumor and antiangiogenic properties, acting through the binding to β-tubulin near the colchicine-binding site. In fact, 2ME2 inhibited microtubule polymerization and induced mitotic arrest [17,18].

In another series of steroids, pregnenolone 20-oxime derivatives showed relevant activity as CYP450_17α_ and 5α-R enzyme inhibitors [19,20,21,22]. The introduction of an oxime group at C6 in androstane series was explored in the development of aromatase inhibitors [23].

Concerning antiproliferative assays, previous studies showed that the presence of oximes in the steroid scaffold originated compounds with relevant potential anticancer interest. For example, 6*E*-hydroxyimino cholest-4-ene derivatives isolated from marine sponges showed relevant antiproliferative activities against several types of cancer cells [4,5]. Later, Cushman et al. developed several 2ME2 analogues with cancer cell growth and tubulin polymerization inhibitory effects. This series of compounds included 2-(2´,2´,2´-trifluoroethoxy)- and 2-ethoxy-6-oxoestradiol as well as their corresponding oximes, which demonstrated clearly higher antiproliferative effects than the ketone analogues in several cancer cell lines, including breast, prostate and colon tumoral cells. In addition, these compounds also inhibited tubulin polymerization and had low binding affinities to ERα [17,24]. In estrane steroids, Rzheznikov and co-workers [25] synthesized 9α-hydroxy,11β-nitrooxyestrone-17-oxime and evidenced in vivo its antitumor effect against breast cancers. However, when compared with the corresponding 17-ketone analogue, the presence of the oxime group seemed to have low influence in this activity. In addition, these two compounds stimulated the tumor growth by the end of a 15-day treatment course, possibly due to their estrogenic effects [25]. Concerning substituted oximes, a large number of estrone-16-oxime ethers were synthesized and their antiproliferative effects were in vitro evaluated against several cell lines. Of these, among other compounds, interesting results were observed for 3-benzyloxy-16-propionyloximino-13α-methylestrone and 3-(4-methoxybenzyloxy)-16-methoximinoestrone as well as for the unsubstituted oximes 16-oximinoestrone and 3-sulfamoyloxy-16-oximinoestrone [15]. Sánchez-Sánchez et al. [26] also evidenced the antitumor effects of steroidal sapogenin oximes on HeLa cell lines and showed that the antiproliferative activity was 2.3–2.8 times higher than the observed with diosgenin. More recently, C20 oxime ester derivatives were prepared from 16-dehydropregnenolone acetate and showed cytotoxicity against leukemia (NB4), prostate (PC-3) and HeLa cancer cells [27].

In view of the therapeutic importance of steroidal oximes, and considering our interest in developing modified estrane derivatives as anticancer agents [7,28], the present study focuses on the synthesis and antiproliferative evaluation of new E1 derivatives bearing an oxime group at C17. Their cytotoxic activities were tested using breast (MCF-7, T47D), prostate (LNCaP), liver (HepaRG), colon (Caco-2) and normal fibroblast (NHDF) cell lines. For the most promising compounds the IC_50_ was determined and then an estrogenicity assay, cell cycle analysis by flow cytometry after propidium iodide staining and fluorescence microscopy using Hoechst 3358 were performed. Molecular docking studies against the ERα, ST, 17β-HSD1 and β-tubulin were also accomplished.

## 2. Results

### 2.1. Chemistry

**Scheme 1:**
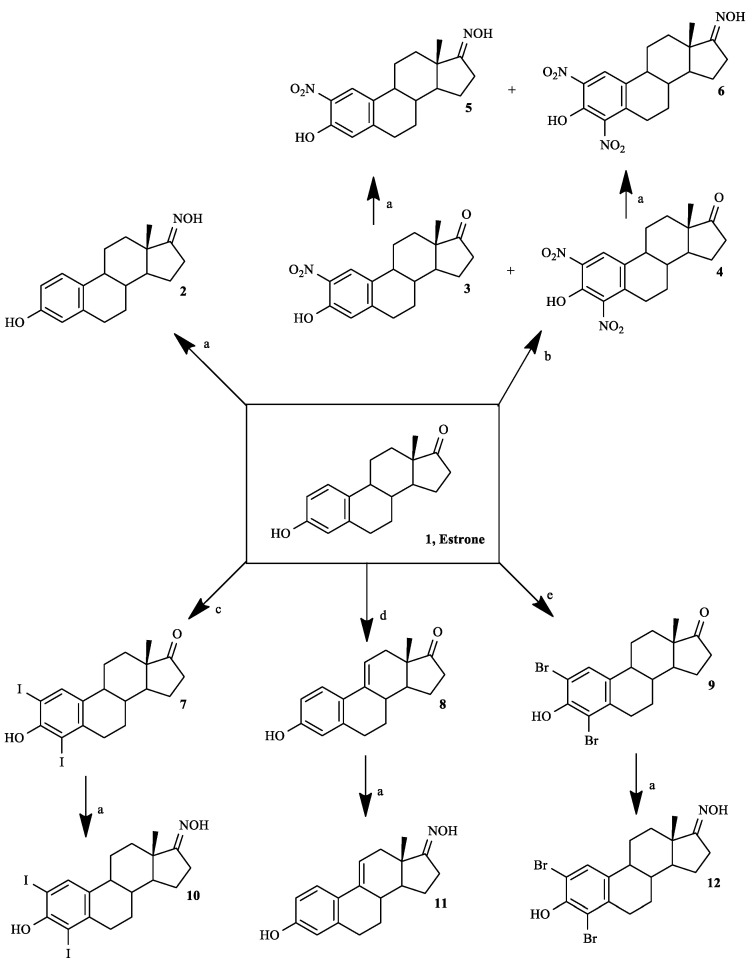
Synthetic route to prepare estrone oxime derivatives. Reagents and conditions: (**a**) NH_2_OH·HCl, NaOH, EtOH, H_2_O, reflux; (**b**) HNO_3_, AcOH, T = 50 °C; (**c**) I_2_, morpholine, PhH, rt; (**d**) DDQ, MeOH, reflux; (**e**) *N*-bromosuccinimide, EtOH, rt.

Six steroidal oximes in estrane series were synthesized as shown in Scheme 1, five of which for the first time (compounds **5, 6**, **10–12**), to the best of our knowledge. All compounds were characterized by spectral analysis (IR, ^1^H- and ^13^C-NMR; in Supplementary Materials) and HRMS was also obtained for the new prepared steroidal oximes. All spectral data are in agreement with the presented structures. The NOH signal in ^1^H-NMR appeared near 10 ppm. In ^13^C-NMR spectra, the signal of C17-ketone appeared near 220 ppm and the C17-NOH near 168–172 ppm. The presence of Δ^9,11^ double bond (compounds **8** and **11**) was associated to the signal of C11-H that appeared at 6.06 in the ^1^H-NMR spectrum [29].

The nitration reaction was performed as described by Stubenrauch et al. [30], which was applied by these authors to obtain 2-nitroestrone **3**. However, as an excess of the nitrating agent was used, 2,4-dinitroestrone **4** was also formed and the mixture of products was separated by column chromatography. The yields of these nitro-steroids were similar to the previously described ones [30]. The introduction of halogens (compounds **7** and **9**) and of Δ^9,11^ double bond (compound **8**) were effected using methodologies already applied by us [28]. Finally, for the preparation of oximes we selected a method involving the use of EtOH, NaOH and hydroxylamine hydrochloride [8] as this is a more selective and greener strategy than other approaches that use, for example, pyridine [25]. In fact, these methods involve more toxic reagents/solvents, are more time consuming, have complex workups and can lead to lower reaction yields [31,32,33].

### 2.2. Biological Testing

#### 2.2.1. Cell Proliferation Studies

All compounds were in vitro tested on MCF-7, T47D, LNCaP, HepaRG, Caco-2 and NHDF cell lines by the MTT colorimetric assay. In this context, it is important to mention that the results for non-oxime compounds **7–9** were described in our previous research work [28].

**Figure 1:**
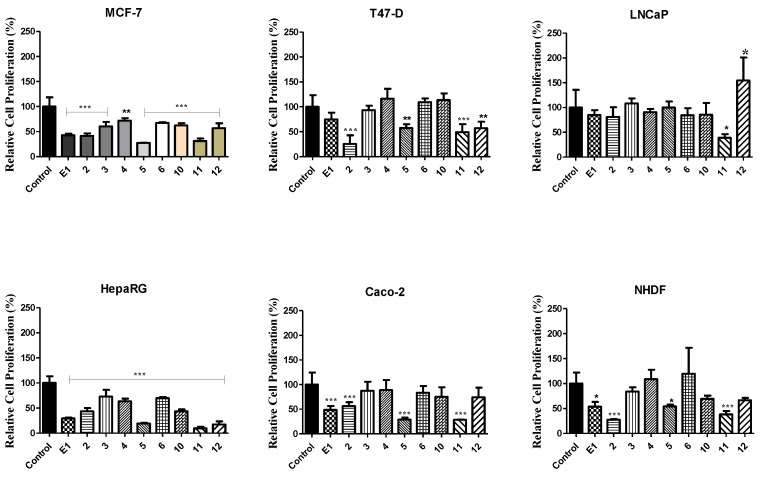
Relative cell proliferation of hormone-dependent (MCF-7, T47-D, LNCaP) and hormone-independent cancer cells (HepaRG, Caco-2 and NHDF) incubated for 72 h at 30 µM with the synthesized compounds, determined by the MTT assay, spectrophotometrically quantifying formazan at 570 nm. Data are expressed as a percentage of cell proliferation relative to the negative control, are indicated as means ± SD and are representative of at least two independent experiments. *** *p* < 0.001, ** *p* < 0.01, * *p* < 0.05 vs. control (the Student t-test).

Firstly, a screening study was performed at 30 µM for a first analysis of the cytotoxic effect of these compounds (Figure 1). This screening showed that several oximes led to a higher reduction in cell proliferation than the observed with parent compounds, which was particularly evident for compounds **5** and **11** in most cell lines. In addition, these two compounds and oxime **2** were the most cytotoxic in these experimental conditions. Furthermore, the cell lines mostly affected by all compounds were MCF-7 and HepaRG. On the other hand, only compound **11** promoted a significant reduction of LNCaP cells proliferation.

After the screening, for the cases where a reduction of cell proliferation was higher than 50%, the IC_50_ was determined (Table 1). Generally, the estimated IC_50_ values were in agreement with the results observed in the screening, confirming that the most potent compounds were oximes **2**, **5** and **11**. Of these, the most cytotoxic was Δ^9,11^-estrone oxime (compound **11**) on LNCaP cells (IC_50_ = 3.59 µM). In addition, the highest selectivity index was also observed for this derivative in these cells (Table 2). However, the variability of MTT assays was higher in LNCaP cells, as we and others have experienced with this poorly adherent cell line, and therefore the fit was less good and the uncertainty is higher in this case.

Although the structure of E1 oxime (compound **2**) is widely known [8,9], few studies concerning its biological activity have been published so far. Interestingly, our data showed a good antiproliferative activity of this compound against HepaRG cells (IC_50_ = 16.94 µM). When evaluating the effect of the presence of Δ^9,11^ double bond (compound **11** vs. compound **2**), it is interesting to note that the effect depends on the cell line, being observed a higher cytotoxicity for compound **11** in MCF-7, LNCaP, Caco-2 and NHDF cells. Concerning the effect of A-ring modifications in these oximes, the introduction of 2-nitro group (compound **5**) allowed an improvement of the cytotoxicity when comparing with its absence (steroid **2**) in MCF-7, HepaRG and Caco-2 cells. In the other hand, the iodination and bromination led to a lower cytotoxicity than the non-functionalized A-ring (compound **2**) and 2-nitroestrone oxime **5**. However, similarly to our previously published results with 2,4-diiodo- and 2,4-dibromoestrone [28], interesting IC_50_ values were observed for A-ring halogenated E1 oximes **10** and **12** on HepaRG cancer cells. In this context, it is important to mention that the nitro group(s) are susceptible to reduction by nitro reductases and the Δ^9,11^ bond of compound **11** is prone to oxidation by the CYP P450 family, which reinforces the importance of study its cytotoxic effect in hepatic cells [34]. On the other hand, 2,4-dinitroestrone oxime (compound **6**) displayed low antiproliferative activity. Finally, considering the data on Figure 1 and Table 1 and Table 2, it is clear that the majority of these compounds had higher cytotoxicity against tumoral (MCF-7, T47D, LNCaP, HepaRG and Caco-2) than non-tumoral (NHDF) cells.

**Figure 2:**
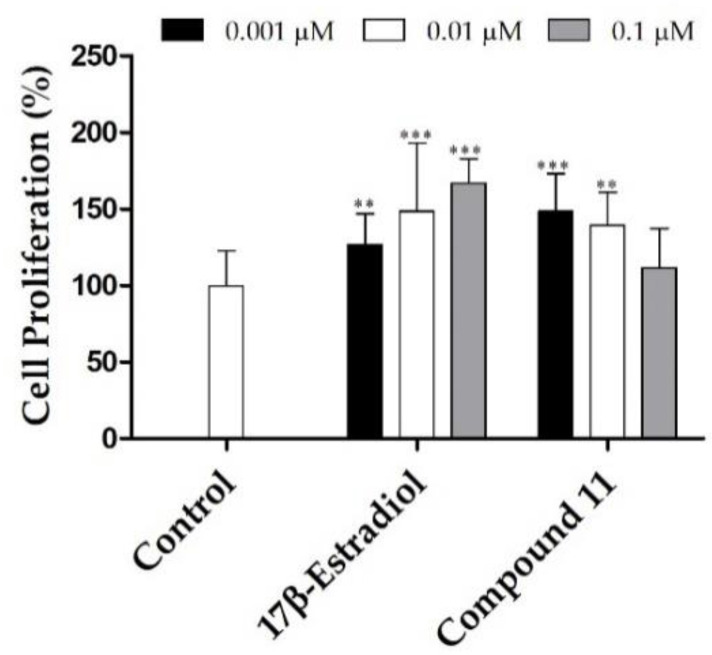
Proliferation of estrogen-sensitive T47-D cells incubated with 17β-estradiol and compound **11** for 24 h. Each bar represents the mean ± SD (originated from two independent experiments). *** *p* < 0.001, ** *p* < 0.01 vs. control (one way ANOVA post-hoc Bonferroni test).

To determine the potential estrogenic profile of the synthesized compound with the most relevant anti-proliferative activity (steroid **11**), its cell growing effect was measured on the estrogen-sensitive breast cancer T47-D cells (ER^+^) in serum-free culture medium. This proliferative/estrogenic activity was expressed as the difference between the cell proliferation (in percentage) caused by a given compound and the basal cell proliferation fixed at 100% (Figure 2) [35,36]. E2 was also tested as reference compound. Unfortunately, similarly to the observed with E2, compound **11** also stimulated the cell proliferation at 0.001 and 0.01 µM when compared with the negative control.

In our previous study, it was also evidenced that Δ^9,11^-E1 (compound **8**) also stimulated the proliferation of T47-D cells [28] and therefore, its conversion into the oxime analogue did not eliminate this effect. In this context, Palomino et al., [37] using X-ray crystallography and molecular modeling studies, showed that the presence of Δ^9,11^ double bond caused a flattening of B, C and D rings and consequently reduced the binding to ER in 1/5^th^ in comparison with E2. Despite this, as evidenced by our results, the presence of C9 = C11 double bond did not eliminate the estrogenic effect characteristic of these compounds. In this context, other reports also showed that the presence of an oxime group did not eliminate this effect. In fact, OMATE had a stimulatory effect (0.15 g ± 0.01) on the uterine growth in ovariectomized rats, which was approximately 50% higher than that of EMATE (0.11 g ± 0.02) [8]. In addition, and as previously referred, despite that 9α-hydroxy,11β-nitrooxyestrone-17-oxime had relevant anti-breast cancer effects, this compound also stimulated the tumor growth after a 15-day treatment period [25]. This preliminary study seemed to suggest the estrogenic activity of compound **11**. Further studies will be necessary to elucidate this activity (e.g., using a luciferase assay) [38].

#### 2.2.2. Cell Survival, Cell Cycle Distribution Evaluation and Hoechst 33,258 Staining

**Figure 3:**
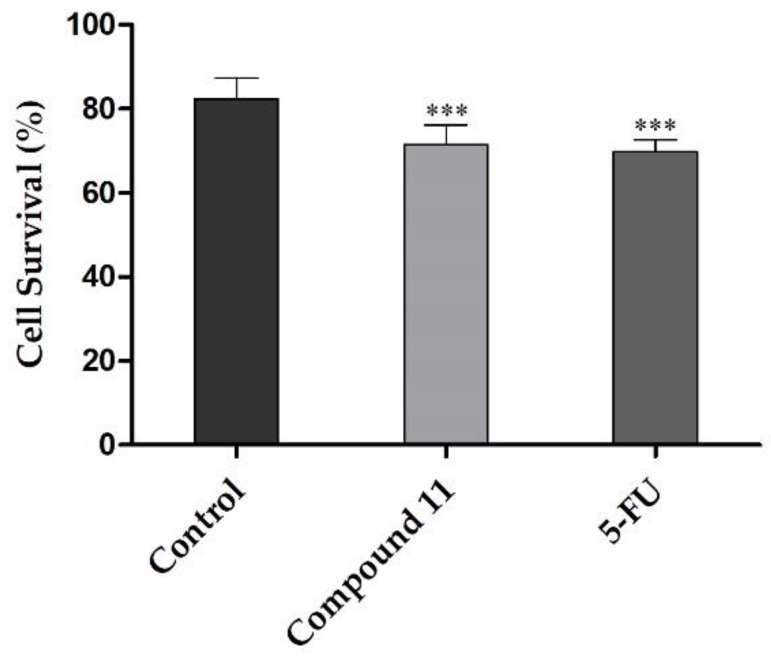
Percentage of LNCaP viable cells after 24 h treatment with 50 µM of compound **11** evaluated through propidium iodide flow cytometry assay. Control corresponds to untreated cells and 5-FU (50 µM) was used for comparison. The percentage of survival is the percentage of cells in R_1_ (live cells) as compared to the total number of events in R_2_ (dead cells) and R_3_ (undetermined cells). Each bar represents the mean ± SD (originated from two independent experiments). *** *p* < 0.001 vs. control (one way ANOVA post-hoc Bonferroni test).

**Figure 4:**
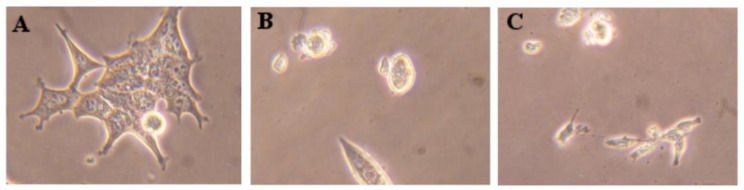
Photographs of the LNCaP cells (**A**, control) treated with 50 µM of compound **11** (**B**) and 5-FU (**C**) for 24 h. Amplification of 100x.

The possible mechanism of action of compound **11** was studied by flow cytometry after PI staining. This assay was performed in LNCaP cells at 24 h post treatment, and 5-FU was used as the positive control. In this cell line, it was observed that compound **11** led to 11% reduction in cell viability (Figure 3). This effect was similar to the one originated by 5-FU (12%). In addition to this flow cytometry study, cells were also observed using an optic microscope (Figure 4) and, after 24 h of treatment with compound **11**, it was possible to see small modifications in LNCaP cells, which lost their shape, becoming rounded, as it is characteristic to happen during mitosis.

**Figure 5:**
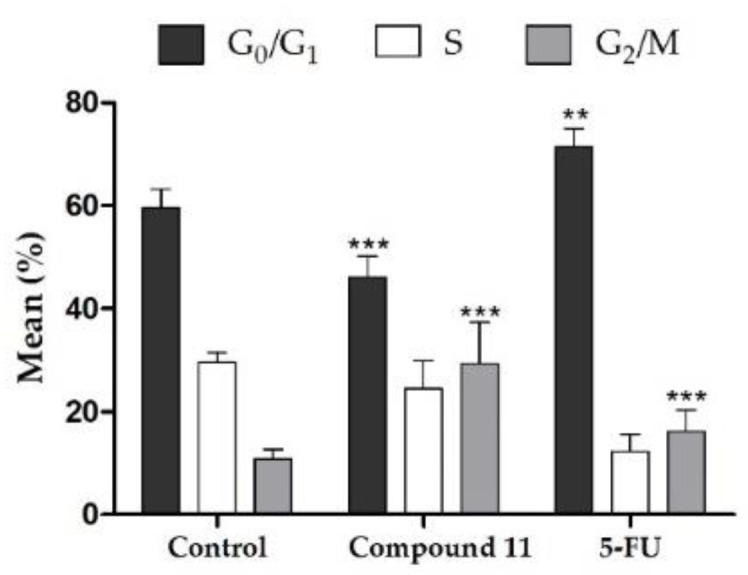
Cell cycle distribution analysis of LNCaP cancer cells after treatment with compound **11** at 50 µM for 24 h. Each bar represents the mean ± SD of four samples (originating from two independent experiments). ** *p* < 0.01 vs. control; *** *p* < 0.001 vs. control (one way ANOVA post-hoc Bonferroni test).

The arrest of cell cycle progression is one of the strategies used to stop cancer proliferation [39]. In this context, some studies previously published showed the effect of steroidal and non-steroidal oximes in cell cycle [26,40]. As example, it was evidenced that E1-16-oxime ethers promoted the apoptotic HeLa cell death and modulated the cell cycle progression (arrest at G_1_), leading to an increase in cellular shrinkage, nuclear condensation, membrane permeability, sub-diploid population and caspase-3 activity [15]. In addition, 16β-triazolyl-17α-estradiol 3-benzyl ethers of the 13α-E2 series led to a G_2_/M cell cycle arrest and caspases-3 and 9 activation [41] and Δ^9,11^-E1 induced an arrest at G_0_/G_1_ in HepaRG cell cycle [28]. Thus, the interference of compound **11** in cell cycle distribution was also evaluated by flow cytometry. Interestingly, it was found that the treatment with this steroid oxime (50 µM, 24 h) induced a G_2_/M cell cycle arrest of LNCaP cells (Figure 5). Also, LNCaP cells were not able to pass through to the S and G_2_/M phases treated by compound 5-FU, which is in agreement with literature for these cells [42].

**Figure 6:**
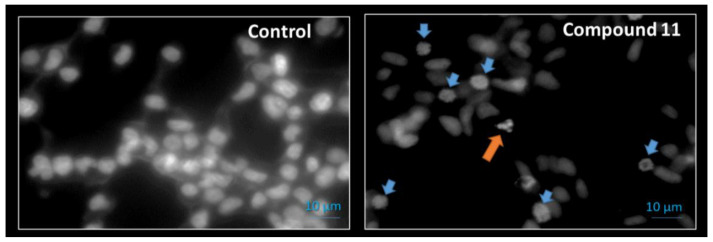
Fluorescence microscopy images of DNA staining (Hoechst 33258) in LNCaP cells treated with vehicle (Control) or with compound **11** at 50 µM for 24 h. Blue down-pointing arrows: condensed DNA onto visible chromosomes (prophase); Orange up-pointing arrow: condensed and fragmented nuclei (apoptosis).

Apoptosis is essential for maintaining the physiologic balance between cell death and cell growth [43]. Therefore, studies for understanding the cancer cell cycle, particularly the interplay with chromatin control, are providing opportunities for developing a new range of anti-cancer drugs [44]. In this context, using a preliminary assay, the Hoechst 33,258 fluorescent dye was used by us to analyze nuclei morphology of LNCaP cells by fluorescence microscopy after exposition to 50 µM of compound **11** during 24 h (Figure 6). Interestingly, it was observed the presence of condensed DNA, typical of prophase, and a small proportion of condensed and fragmented nuclei, typical of apoptosis [45]. β-Tubulin is a protein that polymerize into microtubules, which are involved in cell movement, intracellular trafficking and mitosis [46]. Tubulin-binding drugs, such as paclitaxel and docetaxel [47], kill cancerous cells by inhibiting microtubule dynamics leading to mitotic arrest and cell death. In this context, as it was observed that compound **11** promoted a cell cycle arrest at G_2_/M (Figure 5) and also led to the formation of condensed DNA typical of prophase, plus condensed and fragmented nuclei typical of apoptosis (Figure 6), it can be speculated that it can act by interference with β-tubulin, similarly to which occurs with other steroids of the estrane series like as estramustine and 2ME2 [17,24,48]. In the next section, we studied the interaction of compound **11** and β-tubulin by docking assay to try to better understand the cell cycle arrest.

### 2.3. Molecular Docking Studies

Taking into account the enzymes involved in steroidogenesis, and given the structural similarity of the compounds of our study and several of the above referred steroidal oximes acting by interaction with the mentioned targets, we aimed to evaluate the affinities of the steroids prepared by us and the proteins ERα, ST, 17β-HSD1 and β-tubulin. Molecular docking is a standard computational tool that has been successfully employed in drug design and discovery studies. Satisfactory docking results can be obtained when relatively small ligands with few rotatable bonds are docked towards protein binding pockets in which flexibility does not play an important role. However, for complex molecules (with many rotatable bonds and flexibility), the use of methodology involving theoretical docking and molecular dynamics techniques are important to overtake these limitations, because they allow for evaluating and selecting the best molecule generated in the molecular docking, which can affect the results [49,50,51]. The three-dimensional structural coordinates of these three protein receptors were obtained from PDB, and molecular docking was performed using the program AutoDock vina. To validate the standard docking method, simulations were carried out between crystallized ligands/drugs with the respective proteins and all control redocking simulations were able to reproduce the ligand-protein interaction geometries presented in the respective crystal structures with a RMSD ≤ 2.0 Å. Then, all compounds were docked for the referred targets, as observed in Table 3.

**Figure 7:**
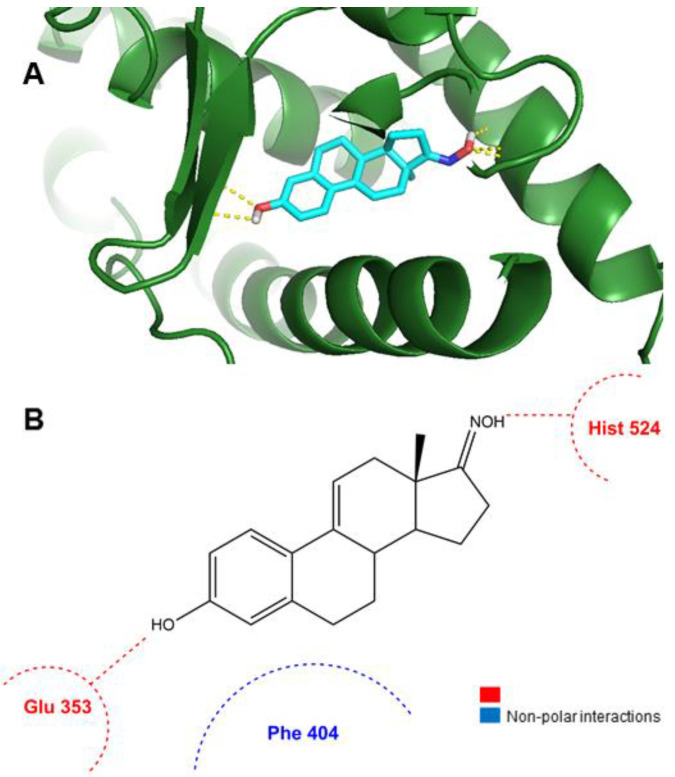
Analysis of predicted ERα binding orientations for the best raking compound, **11**. (**A**) 3D molecular and (**B**) 2D docking results showing the main interactions.

The results observed in the docking simulations with ERα, ST and 17β-HSD1 for compounds **7–9** are presented in our previous work [28]. According to the data presented in Table 3, compound **11** can bind ERα in a lower energy than the control (E2). In addition, in Figure 7 can be observed that this compound can form two hydrogen bonds between its oxime group at C17 and Hist 524 and between the hydroxyl group at C3 and Glu 353 of ERα target. These interactions are similar to the observed with E2. Therefore, globally, these docking data seem to be in agreement with our experimental results (Figure 2).

**Figure 8:**
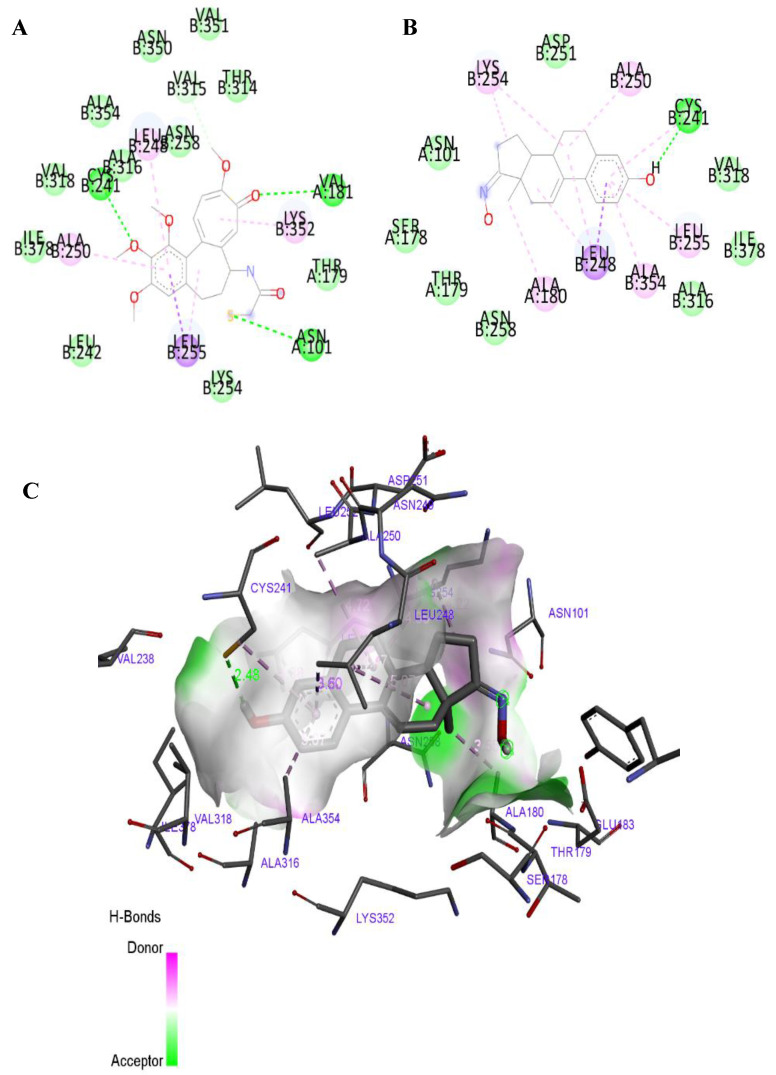
2D diagram of the active interaction between colchicine and β-tubulin (panel A). 2D diagram of predicted interactions between best ranked compound, **11**, and β-tubulin (panel B) and 3D representation (panel C). (**A**,**B**) Conventional hydrogen bonds are displayed in green, Van der Waals interactions in light green, Pi-sigma interactions are displayed in purple and alkyl an Pi-alkyl interactions in pink. (**C**) All contact and hydrogen bonds are shown as dotted lines, and represented with same colors above described.

The predicted affinity energies of the synthesized compounds for ST are all higher than the energy obtained for co-crystalized ligand (Table 3). This suggested that these compounds have a poor affinity to this macromolecule. Concerning the energy values obtained in the docking studies with 17β-HSD1, generally they were very close to the determined affinity of the co-crystalized ligand (5α-dihydrotestosterone, DHT) (Table 3). Due to these interesting results, we also analyzed the interaction mode of the best ranked compounds, **5** and **11**, with 17β-HSD1. It is already known from the literature that the main interactions between DHT and this enzyme are a conventional hydrogen bond with Hist 221 residue and Van der Waals interactions with Leu 149, Val 143 and Pro 187 residues [52]. However, the studied compounds just exhibited the Van der Waals interactions, lacking the hydrogen bond with Hist221, which can be determinant for their interaction with this target. Further in vitro studies will be necessary to elucidate the significance of this interaction. The most interesting binding was observed between compound **11** and β-tubulin, as shown in Figure 8 and Table 3. Besides the good affinity energy value, which was lower than the determined for colchicine, compound **11** was also predicted to have the most important interactions with β-tubulin, such as the conventional hydrogen bond with Cys B 241, alkyl and Pi-alkyl interactions with Leu B 248, and Van der Waals interaction with Val B 318 [53,54,55]. Interestingly, previous studies suggested that the tubulin ligand interactions through amino acid residues Ala316 and Val318 are very crucial in inducing antitubulin effect [55]. The other studied compounds, despite having good binding energies, did not establish the conventional bonds to tubulin. Compounds **8** and **9** did not show a conventional hydrogen bond with Cys B 241, interacting only through Van der Waals, alkyl and Pi-alkyl interactions, which explain that the affinity energies may not be directly related to the established interactions required with the active site. Therefore, the cell cycle arrest at G_2_/M originated by compound **11** (Figure 5), at prophase (Figure 6), can perhaps occur due to β-tubulin inhibition. However, future studies are needed to prove this hypothesis.

## 3. Materials and Methods

### 3.1. Chemistry

All chemicals received from suppliers were used without further purification. The following reagents were purchased from: E1: Cayman Chemical (Ann Arbor, MI, USA); hydroxylamine hydrochloride: Fluka (Buchs, Switzerland; methanol (MeOH): Fisher Chemical (Hampton, MA, USA); *N*-bromosuccinimide (NBS): Alfa Aesar (Haverhill, MS, USA); ethanol (EtOH) 99.9%: Manuel Vieira & Cª (Torres Novas, Portugal). In addition, 2,3-dichloro-5,6-dicyano-*p*-benzoquinone (DDQ), morpholine, 17β-estradiol (E2), and dimethyl sulfoxide (DMSO) as well as the remaining chemical products referred in the text were obtained from Sigma-Aldrich (St. Louis, MO, USA). Deuterated DMSO (DMSO-*d*_6_) and deuterated chloroform (CDCl_3_) were purchased from Armar Chemicals (Leipzig, Germany). All reactions were monitored by thin layer chromatography (TLC) using Al-backed aluminum/silica gel plate 0.20 mm (Macherey-Nagel 60 F254, Duren, Germany). After elution, plates were visualized in a CN-15.LC UV chamber under ultraviolet (UV) radiation (254 nm). Then, the EtOH/concentrated sulfuric acid (95:5, v:v) solution was used, followed by heating at 120 °C, to reveal the plates. The evaporation of solvents was achieved by using a rotary vacuum drier from Büchi (R-215). Infrared (IR) spectra were collected on a Thermoscientific Nicolet iS10 equipped with a diamond attenuated total reflectance crystal at room temperature in the 4000–400 cm^−1^ range by averaging 16 scans at a spectral resolution of 2 cm^−1^. Nuclear magnetic resonance (NMR) spectra (^1^H-NMR and ^13^C-NMR) were acquired on a Bruker Avance 400 MHz spectrometer and were processed with the software TOPSPIN 4.07 (Bruker, Fitchburg, WI, USA). Chemical shifts are reported in parts per million (ppm) relative to tetramethylsilane (TMS) or solvent as an internal standard. Coupling constants (*J* values) are reported in hertz (Hz) and splitting multiplicities are described as s = singlet; brs = broad singlet; d = doublet and dd = double doublet. High resolution mass spectrometry (ESI-HRMS) was performed by the microanalysis service on a QSTAR XL instrument (Salamanca, Spain).

#### 3.1.1. Procedures for the Synthesis of Intermediates—Compounds **3**, **4**, **7**, **8** and **9**

The intermediates **7**, **8** and **9** were synthesized and structurally characterized as previously described [28].

##### Synthesis of 3-hydroxy-2-nitroestra-1,3,5(10)-trien-17-one (3) and 3-hydroxy-2,4-dinitroestra-1,3,5(10)-trien-17-one (**4**)

E1 (541 mg, 2 mmol) was added to 16.3 mL of glacial acetic acid and the mixture was vigorous stirred at 50 °C. Then, a solution of nitric acid 70% (178 µL) in glacial acetic acid (540.6 µL) was added dropwise and the mixture was stirred at room temperature for 48 h. After completion (TLC control), the reaction mixture was diluted in 150 mL of ethyl acetate and the resulting solution was washed with 50 mL of saturated NaCl solution, 50 mL of saturated NaHCO_3_ solution and 50 mL of H_2_O. Next, the solution was dried over anhydrous Na_2_SO_4_, filtered and concentrated under reduced pressure to yield the crude product, which was purified by column chromatography (eluent: gradient of ethyl acetate (EA)/petroleum ether (PE) 40–60 °C, 1:3; 1:1 and 3:1) to give compound 3 as yellow solid (261 mg, 41%) and compound 4 as orange solid (341 mg, 47%) [30,56,57].

##### 3-Hydroxy-2-nitroestra-1,3,5(10)-trien-17-one (**3**)

IR (ʋ_max_/cm^−1^): 896, 1258, 1306, 1372, 1429, 1478, 1519, 1563, 1628, 1733, 2858–2932, 3040, 3295; ^1^H-NMR (400 MHz, CDCl_3_) δ: 0.89 (s, 3H, C18-CH_3_), 6.84 (s, 1H, C4-H), 7.96 (d, 1H, *J* = 1.1 Hz, C1-H), 10.39 (s, 1H, OH); ^13^C-NMR (100 MHz, CDCl_3_) δ: 13.97, 21.75, 25.90, 26.10, 29.83, 31.47, 35.98, 37.93, 43.66, 48.02, 50.56, 119.18, 121.77, 131.98, 133.31, 149.00, 153.12, 220.37.

##### 3-Hydroxy-2,4-dinitroestra-1,3,5(10)-trien-17-one (**4**)

IR (ʋ_max_/cm^−1^): 902, 1246, 1376, 1455, 1532, 1571, 1629, 1729, 2872–2939, 3185; ^1^H-NMR (400 MHz, CDCl_3_) δ: 0.90 (s, 3H, C18-CH_3_), 8.14 (d, 1H, *J* = 1.0 Hz, C1-H), 10.60 (s, 1H, OH); ^13^C-NMR (100 MHz, CDCl_3_) δ: 13.91, 21.64, 24.95, 25.06, 25.98, 31.33, 35.89, 37.21, 43.65, 47.85, 50.23, 122.87, 132.33, 133.72, 139.29, 141.87, 145.06, 219.76.

#### 3.1.2. General Procedure for the Synthesis of Oximes

To a solution of parent compound in EtOH (8.1 mL/1 mmol of parent compound) were added hydroxylamine hydrochloride (2.9 mmol), NaOH (2 mmol) and water (272 µL). The mixture was heated under reflux for 3 h and upon cooling poured into an aqueous solution of 1 N HCl (2.7 mL). The formed precipitate was filtered, washed with cold water, and air-dried to give the corresponding product. After, the solid was recrystallized using MeOH to afford the pure product [8]. As compounds **6** and **12** did not precipitate after addition to HCl solution, the work up was performed as described below.

##### Synthesis of 17-hydroxyiminoestra-1,3,5(10)-trien-3-ol (**2**)

Compound 2 was prepared from compound 1 (270.37 mg, 1 mmol) to give white solid (265 mg; 93%). After recrystallization compound 2 was obtained as colorless crystals [8]. IR (ʋ_max_/cm^−1^): 869, 918, 1151, 1237, 1283, 1348, 1373, 1460, 1497, 1583, 1618, 2868–2929, 3025, 3252, 3405; ^1^H-NMR (400 MHz, DMSO-*d_6_*) δ: 0.85 (s, 3H, C18-CH_3_), 6.44 (d, 1H, *J* = 2.5 Hz, C4-H), 6.51 (dd, 1H, *J_1_* = 8.5 Hz, *J_2_* = 2.5 Hz, C2-H), 7.04 (d, 1H, *J* = 8.5 Hz, C1-H), 8.99 (brs, 1H, C3-OH), 10.08 (brs, 1H, NOH); ^13^C-NMR (100 MHz, DMSO-*d_6_*) δ: 17.32, 22.51, 24.89, 25.92, 26.84, 29.08, 34.27, 37.87, 43.36, 43.59, 52.49, 112.75, 114.94, 125.99, 130.15, 137.08, 154.96, 167.95.

##### Synthesis of 17-hydroxyimino-2-nitroestra-1,3,5(10)-trien-3-ol (**5**)

Compound 5 was prepared from compound 3 (157.68 mg, 0.5 mmol) to give a yellow solid (120 mg, 73%). After recrystallization from MeOH, compound 5 was obtained as yellow crystals. IR (ʋ_max_/cm^−1^): 927, 1017, 1095, 1259, 1298, 1373, 1433 1482, 1518, 1579, 1633, 2858–2961, 3283; ^1^H-NMR (400 MHz, DMSO-*d_6_*) δ: 0.85 (s, 3H, C18-CH_3_), 6.83 (s, 1H, C4-H), 7.75 (s, 1H, C1-H), 10.11 (s, 1H, C3-OH), 10.54 (brs, 1H, NOH); ^13^C-NMR (100 MHz, DMSO-*d_6_*) δ: 17.21, 22.46, 24.87, 25.52, 26.11, 28.83, 33.97, 37.04, 42.86, 43.23, 52.34, 118.49, 121.45, 131.91, 134.07, 145.96, 150.25, 167.74. HRMS (ESI-TOF): *m/z* [M^+^ + H] calcd for C_18_H_22_N_2_O_4_: 330.1580; found 330.1573.

##### Synthesis of 17-hydroxyimino-2,4-dinitroestra-1,3,5(10)-trien-3-ol (**6**)

Compound 6 was prepared from compound 4 (180.04 mg, 0.5 mmol). As the crude did not precipitate after the addition of HCl, the workup was performed in a different manner. For this, the reactional mixture was diluted in 100 mL of dichloromethane and washed with 50 mL of saturated NaCl solution, 50 mL of saturated NaHCO_3_ solution and 50 mL of H_2_O, dried over anhydrous Na_2_SO_4_, filtered and concentrated under reduced pressure. For analysis and use in cell studies, a sample was recrystallized from MeOH to afford compound 6 as dark yellow crystals (130.7 mg, 73%). IR (ʋ_max_/cm^-^1): 927, 1025, 1184, 1259, 1305, 1356, 1377, 1436, 1468, 1537, 1577, 1630, 2931, 3240, 3612; ^1^H-NMR (400 MHz, DMSO-*d_6_*) δ: 0.86 (s, 3H, C18-CH_3_), 7.99 (s, 1H, C4-H), 10.12 (s, 1H, NOH); ^13^C-NMR (100 MHz, DMSO-*d_6_*) δ: 17.17, 22.39, 23.94, 24.85, 24.96, 25.43, 33.87, 36.14, 42.76, 43.17, 52.01, 122.79, 133.01, 135.25, 135.99, 141.98, 142.92, 167.62. HRMS (ESI-TOF): *m/z* [M^+^ + H] calcd for C_18_H_21_N_3_O_6_: 375.1430; found 375.1424.

##### Synthesis of 17-hydroxyimino-2,4-diiodooestra-1,3,5(10)-trien-3-ol (**10**)

Compound 10 was prepared from compound 7 (99.06 mg, 0.19 mmol) to give a beige solid (69 mg, 34%). IR (ʋ_max_/cm^−1^): 800, 926, 1027, 1098, 1170, 1262, 1293, 1327, 1388, 1450, 2864–2927, 3270, 3451; ^1^H-NMR (400 MHz, CDCl_3_) δ: 0.94 (s, 3H, C18-CH_3_), 7.59 (s, 1H, C1-H); ^13^C-NMR (100 MHz, CDCl_3_) δ: 17.23, 23.02, 25.75, 26.63, 28.13, 33.95, 37.28, 37.29, 44.01, 44.54, 52.88, 78.57, 92.24, 136.01, 136.28, 140.82, 151.64, 172.12. HRMS (ESI-TOF): *m/z* [M^+^ + H] calcd for C_18_H_21_I_2_NO_2_: 536.9662; found 536.9632.

##### Synthesis of 17-hydroxyimino-3-hydroxyestra-1,3,5(10),9(11)-tetraen-3-ol (**11**)

Compound 11 was obtained from compound 8 (67.1 mg, 0.25 mmol) to give a white solid (55.2 mg, 78%). IR (ʋ_max_/cm^−1^): 926, 1155, 1246, 1279, 1354, 1426, 1465, 1494, 1575, 1613, 1627, 2831–2956, 3020, 3342; ^1^H-NMR (400 MHz, DMSO-*d_6_*) δ: 0.86 (s, 3H, C18-CH_3_), 6.06 (m, 1H, C11-H), 6.45 (d, 1H, *J* = 2.5 Hz, C4-H), 6.54 (dd, 1H, *J_1_* = 8.7 Hz, *J_2_* = 2.5 Hz, C2-H), 7.42 (d, 1H, J = 8.7, C1-H), 9.25 (br s, 1H, OH); 10.18 (br s, 1H, NOH). ^13^C-NMR (100 MHz, DMSO-*d_6_*) δ: 18.03, 23.56, 25.32, 27.98, 29.32, 36.89, 37.47, 41.59, 49.88, 113.86, 114.87, 116.04, 125.11, 125.29, 135.18, 137.18, 156.18, 168.05. HRMS (ESI-TOF): *m/z* [M^+^ + H] calcd for C_18_H_21_NO_2_: 283.1572; found 283.1564.

##### Synthesis of 17-hydroxyimino-2,4-dibromo-3-hydroxyestra-1,3,5(10)-trien-3-ol (**12**)

Compound 12 was obtained from compound 9 (107.4 mg, 0.25 mmol). As the crude did not precipitate after the addition of HCl, the workup was performed in a different manner. For this, the reactional mixture was diluted in 150 mL of dichloromethane and washed with 50 mL of HCl 5%, 50 mL of saturated NaCl solution and 50 mL of H_2_O, dried over anhydrous Na_2_SO_4_, filtered and concentrated under reduced pressure to produce a white solid 12 (95 mg, 86%). IR (ʋ_max_/cm^−1^): 799, 927, 1019, 1097, 1173, 1260, 1398, 1463, 2869–2961, 3273, 3486; ^1^H-NMR (400 MHz, CDCl_3_) δ: 0.93 (s, 3H, C18-CH_3_), 7.38 (s, 1H, C1-H); ^13^C-NMR (100 MHz, CDCl_3_) δ: 17.28, 23.04, 25.55, 26.56, 27.36, 31.13, 33.98, 37.27, 44.10, 44.43, 52.92, 106.64, 113.43, 128.69, 135.41, 136.67, 147.43, 171.72. HRMS (ESI-TOF): *m/z* [M^+^ + H] calcd for C_18_H_21_Br_2_NO_2_: 440.9939; found 440.9932.

### 3.2. Biology

#### 3.2.1. Cell Culture

Breast (MCF-7, T47-D), prostatic (LNCaP) and colon (Caco-2) cancer cells, as well as normal human dermal fibroblasts (NHDF), were obtained from American Type Culture Collection (ATCC; Manassas, VA, USA). The hepatic (HepaRG) cell line was acquired to Life Technologies-Invitrogen™ (through Alfagene, Portugal). They were cultured in 75 cm^2^ culture flasks at 37 °C in a humidified air incubator with 5% CO_2_. MCF-7 cells were maintained with high-glucose Dulbecco’s modified Eagle medium (DMEM) supplemented with 10% fetal bovine serum (FBS; Sigma-Aldrich, St Louis, MO, USA) and 1% antibiotic/antimycotic mixture (10,000 units/mL penicillin G, 100 mg/mL streptomycin and 25 μg/mL amphotericin B) (Sigma-Aldrich, St Louis, MO, USA). High glucose DMEM supplemented with 10% FBS and 1% of the antibiotic mixture of 10,000 units/mL penicillin G and 100 mg/mL of streptomycin (Sigma-Aldrich, St Louis, MO, USA) was used for Caco-2 cells. LNCaP and T47-D cells were cultured in RPMI 1640 medium with 10% FBS and 1% antibiotic mixture. NHDF cells grew in RPMI 1640 medium supplemented with 10% FBS, 2 mM *L*-glutamine, 10 mM HEPES, 1 mM sodium pyruvate and 1% antibiotic/antimycotic. Lastly, hepatic cells were seeded in Williams’ E medium supplemented with 10% FBS, 1% antibiotic mixture, 5 µg/mL insulin, and 5 × 10^−5^ M hydrocortisone hemisuccinate (Sigma–Aldrich, St Louis, MO, USA).

#### 3.2.2. Preparation of Stock Solutions

The stock solutions of compounds were prepared in DMSO at 10 mM and stored at 4–8 °C. From these, the different diluted solutions of compounds were prepared in the corresponding complete culture medium before each experiment. The maximum DMSO concentration in cell studies was 1%.

#### 3.2.3. Antiproliferative Activities against Six Cell Lines

The antiproliferative effect of compounds was evaluated by the 3-(4,5-dimethylthiazol-2-yl)-2,5-diphenyltetrazolium bromide (MTT; Sigma-Aldrich, St Louis, MO, USA) assay in MCF-7, T47-D, LNCaP, HepaRG, Caco-2 and NHDF cells. After reaching near confluence, cells were trypsinized and counted with a hemocytometer by means of the trypan-blue exclusion of dead cells. Then, 100 µL of cell suspension (2 × 10^4^ cells/mL) was seeded in 96-well culture plates and left to adhere and growth during 48 h. After this period, the medium was replaced by solutions of the compounds in study (30 µM for screening assays and 0.1, 1, 10, 25, 50 and 100 µM for concentration-response studies) in the appropriate cell culture medium for approximately 72 h. Then, cells were washed with 100 µL of phosphate buffer saline (PBS; NaCl 137 mM, KCl 2.7 mM, Na_2_HPO_4_ 10 mM and KH_2_PO_4_ 1.8 mM, pH 7.4), and 100 µL of the MTT solution (5 mg/mL), prepared in the appropriate serum-free medium, was added to each well, followed by incubation for approximately 4 h at 37 °C. Afterward, MTT containing medium was removed and formazan crystals were dissolved in DMSO. Absorbance was measured at 570 nm using a microplate reader Bio-rad Xmark spectrophotometer. After background subtraction, cell proliferation values were expressed as percentage relatively to the absorbance determined in negative control cells. Untreated cells were used as the negative control and the clinical drug 5-fluorouracil (5-FU) was used as positive control. Each experiment was performed in quadruplicate and independently repeated.

#### 3.2.4. E-Screening Assay in T47-D Cells

Breast T47-D cells (2 × 10^4^ cells/mL; 100 µL) were seeded in 96-well culture plates in RPMI 1640 medium supplemented with 10% FBS and allowed to attach. After overnight incubation, the medium was replaced every 3 days with fresh phenol red free RPMI 1640 medium supplemented with 5% of dextran-coated charcoal-treated fetal calf serum (DCC-FCS) and containing compound 11 (0.1, 0.01 and 0.001 µM). After 6 days of exposure, the proliferation of T47-D cells was estimated by the MTT assay as described in the previous section. Each experiment was performed in quadruplicate and independently repeated. After background subtraction, cell proliferation values were expressed as percentage relatively to the absorbance determined in negative control cells.

#### 3.2.5. Analysis of LNCaP Cells Viability by Flow Cytometry

The analysis of LNCaP cells viability was performed by flow cytometry after staining with propidium iodide (PI) (solution of PI 1 mg/mL in 0.1% of sodium azide and water; Sigma Aldrich, St Louis, MO, USA). Briefly, 3 mL of cells suspension were seeded in 6-well plates (5 × 10^4^ cells/mL) in complete culture medium. After 48 h they were treated with 50 µM of compound 11. Untreated cells were used as negative control and 5-FU (50 µM) was used as positive control. Each experiment was performed in duplicate and independently repeated. At the end of 24 h of incubation, the supernatant of each well was collected, cells were harvested by trypsinization and pooled with the supernatants. The resulting cell suspension was kept on ice, pelleted by centrifugation and resuspended in 400 µL of complete medium. Afterward, 395 µL of the cell suspension was transferred to a FACS tube and 5 µL of PI and 0.5 µL of ethylenediamine tetraacetic acid (EDTA, 0.123 M) were added. A minimum of 20,000 events was acquired using a BD Accuri C6 (San Jose, CA, USA) flow cytometer in the channels forward scatter (FSC), side scatter (SSC) and fluorescence channel-3 (FL3, for PI). Acquisition and analysis were performed with BD Accuri Software. In the FSC/FL3 contour plot, three regions were created, one corresponding to viable cells (R1), another to dead cells (R2) and a third to an indeterminate cell population between the other two regions (R3) excluding debris that were not considered in the analysis (data not shown). The percentage of viability is the percentage of cells in R1 as compared to the total number of events in R1, R2 and R3.

#### 3.2.6. Cell Cycle Distribution of LNCaP Cells

After 24 h of treatment with compound 11 at 50 µM (6-well plates, 5 × 10^4^ cells/mL), LNCaP cells were collected and washed with PBS and resuspended in 450 µL of a cold solution of 0.5% bovine serum albumin (BSA; Amresco, Solon, OH, USA) and 1 mM EDTA in PBS, followed by fixation with 70% EtOH and kept at −20 °C. Afterward, fixed cells were washed twice with PBS and resuspended in a solution of PI (50 µg/mL) prepared in 0.5% BSA and 1 mM EDTA in PBS and then incubated with Ribonuclease A from bovine pancreas at a final concentration of 0.5 µg/µL (solution in 50% glycerol, 10 mM Tris-HCl, pH 8; Sigma Aldrich, St Louis, MO, USA) for 15 min in the dark. For comparison, untreated cells were used as negative control and cells treated with 5-FU at 50 µM were used as positive control. Each experiment was performed in duplicate and independently repeated. A minimum of 10,000 events was acquired using BD Accuri Software and analysis was performed by Modfit software (Becton Dickinson, San Jose, CA, USA).

#### 3.2.7. Fluorescence Microscopy in LNCaP Cells after DNA Staining

Near-confluent LNCaP cells were seeded in a 6-well plate (5 × 10^4^ cells/mL). After adherence and incubation for 24h with compound 11 (50 µM), the dye Hoechst 33,258 was added to the culture medium to achieve a final concentration of 1µg/mL. The cells were incubated for 15 min at 37 °C and were then photographed by means of a Nikon Eclipse microscope equipped with a fluorescence attachment containing the appropriate optical blocks and a QCapture CCD camera. Apoptosis was revealed by nuclear changes such as chromatin condensation and nuclear fragmentation.

#### 3.2.8. Data Analysis

Data were expressed as mean ± standard deviation (SD). *t*-Student test (two groups) and one-way ANOVA (three groups) were used followed by Bonferroni *post hoc* tests to determine statistically significant differences among the means. Difference between groups was considered statistically significant for a *p*-value lower than 0.05 (*p* < 0.05). The determination of IC_50_ was performed by sigmoidal fitting analysis [log(inhibitor) vs. normalized response—Variable slope], considering a confidence level of 95%.

### 3.3. Molecular Docking Studies

#### 3.3.1. Preparation of Proteins for Molecular Docking

The crystal structures of ERα (PDB ID: 1A52), ST (PDB ID: 1P49), 17β-HSD1 (PDB ID: 3KLM) and β-tubulin (PDB ID: 1SA0) were retrieved from Protein Data Bank [58,59,60,61]. The coordinates of all non-standard residues, including the co-crystalized ligand, were deleted using the software Chimera (v. 1.10.1). Then, non-polar hydrogens were merged in AutoDockTools (v. 1.5.6) and Kollman and Gasteiger partial charges were added. Lastly, the prepared structure was converted from the PDB format to PDBQT for posterior use in the docking simulations.

#### 3.3.2. Preparation of Ligands

All ligands used in docking simulations were built using ChemDraw (v. 12.0) software. Energy minimization and geometry optimization of these molecules were performed in Chem3D (v. 12.0) and the obtained structures were saved as PDB file format. The process of energy minimization was applied in a range from −20 to −40 kcal.mol^−1^. Then, the ligands were completely prepared for docking choosing torsions and the structures were converted into PDBQT format using the software AutoDockTools.

#### 3.3.3. Grid Parameters

The grid parameters were selected using AutoDock vina and AutoDockTools based on the coordinates of the co-crystalized ligand for each case: E2, *N*-acetyl-*D*-glucosamine, 5α-dihydrotestosterone (DHT) and colchicine, with the respective macromolecule. The grid box was centered on the ligand with the following coordinates: for ERα, the coordinates were x = 107.27, y = 13.94, z = 96.38; for ST were x = 62.033, y = −12.215, z = 52.512; for 17β-HSD1 were x = 11.643, y = 9.297, z = −11.887; and for β-tubulin were x = 118.921, y = 89.718, z = 5.932. The size of grid box was 20 × 20 × 20 with 1.0 Å of spacing.

#### 3.3.4. Docking Simulations

After the preparation of macromolecules and ligands, molecular docking simulations were performed using AutoDock vina executable [62], which uses an iterated local search global optimizer. The parameter exhaustiveness of performed experiments was defined as 15. The results of molecular docking were analyzed and visualized in Discovery Studio Visualizer program from BIOVIA software.

#### 3.3.5. Validation of the Molecular Docking Performance

Scoring functions are essential for molecular docking performance. In order to validate the docking performance of AutoDock vina, the difference between the real and best-scored conformations were analyzed by re-docking ERα with E2, ST with *N*-acetyl-*D*-glucosamine, 17β-HSD1 with DHT and β-tubulin with colchicine. Low root-mean-square distance (RMSD) values (<2.0 Å) were obtained for all the four cases, which means that the docking process was reliable and validated [63].

## 4. Conclusions

Several E1 oxime derivatives were synthesized and revealed interesting effects against the proliferation of several tumor cell lines when compared with parent ketone compounds. Of these, oxime **11** showed the highest activity against LNCaP cancer cells, as well as a very relevant selectivity index. In addition, it was also demonstrated that this compound originated cell cycle arrest in G_2_/M on these cells in prophase and condensed and fragmented nuclei characteristic of apoptosis. However, in an *E-screening* assay this oxime also promoted the proliferation of T47-D cells. Docking studies evidenced that compound **11** also showed relevant affinities for ERα and β-tubulin, which could explain its mechanism of action and estrogenic effect. Interestingly, the oximes bearing halogens in A-ring (2,4-diiodoestrone oxime **10** and 2,4-dibromoestrone oxime **12**), evidenced a selectivity for HepaRG cancer cells. Another A-ring functionalized derivative, 2-nitroestrone oxime, but not its 2,4-dinitro analogue, showed higher cytotoxicity against HepaRG and MCF-7 cancer cells. Thus, the presence of an oxime group at C17 in functionalized E1 scaffold was shown to be a good strategy to obtain new molecules with relevant anticancer effects.

## Figures and Tables

**Table 1:** Estimated IC_50_ values (µM) for various compounds in breast (MCF-7, T47-D), prostate (LNcaP), liver (HepaRG), colon (Caco-2) and normal fibroblast cells (NHDF) ^a^.

Compound	MCF-7	T47-D	LNCaP	HepaRG	Caco-2	NHDF
1	41.93	ND	ND	29.53	42.69	61.82
2	26.65	29.26	ND	16.94	ND	43.88
5	16.75	ND	ND	12.32	18.51	ND
10	ND	ND	ND	28.00	ND	ND
11	25.63	43.45	3.59	18.35	24.33	30.84
12	ND	ND	ND	21.94	ND	ND
5-FU	1.71	0.54	7.79	1.78	1.31	3.61

^a^ Cells were treated with different concentrations (0.1, 1, 10, 25, 50 and 100 µM) during 72 h. The cell proliferation effects were determined by the MTT assay. The data shown are representative of at least two independent experiments. 5-FU: 5-fluoruracil. ND: not determined.

**Table 2:** Selectivity index ^a^ of compounds **1**, **2, 11** and 5-fluouracil (5-FU).

Compound	MCF-7	T47-D	LNCaP	HepaRG	Caco-2
1	1.47	ND	ND	2.09	1.45
2	1.65	1.50	ND	2.59	ND
11	1.20	0.71	8.59	1.68	1.27
5-FU	2.11	6.69	0.46	2.03	2.76

^a^ Selectivity index is the ratio of the IC_50_ values of the treatments of non-tumor cells (NHDF) and tumor cells (MCF-7, T47-D, LNCaP, HepaRG and Caco); ND: not determined.

**Table 3:** Predicted affinity energies of compounds **1–12** calculated against the estrogen receptor α (ERα), steroid sulfatase (ST), 17β-hydroxysteroid dehydrogenase type 1 (17β-HSD1) and β-tubulin by AutodockTools with vina executable. Binding energies of co-crystalized ligand in the X-ray crystal structures were calculated by re-docking.

Compound	Lowest Energy (kcal.mol^−1^)			
	ERα	ST	17β−HSD1	β−Tubulin
1	−10.3 ^b^	−6.2 ^b^	−8.1 ^b^	−9.0
2	−9.7	−6.7	−8.1	−8.9
3	−8.8	−7.0	−8.2	−8.7
4	−6.6	−7.0	−8.1	−8.5
5	−7.9	−6.9	−8.3	−8.9
6	−5.3	−6.8	−8.1	−8.4
7	−4.3 ^b^	−6.0 ^b^	−7.7 ^b^	−8.4
8	−10.9 ^b^	−5.9 ^b^	−8.2 ^b^	−9.0
9	−6.8 ^b^	−6.3 ^b^	−8.1 ^b^	−9.0
10	−5.8	−6.6	−7.7	−8.5
11	−10.5	−6.5	−8.3	−9.1
12	−5.0	−6.3	−7.6	−8.5
E2	−9.9 ^a^	−	−	−
*N*−acetyl−*D*−glucosamine	−	−7.2 ^a^	−	−
5α−Dihydrotestosterone	−	−	−8.3 ^a^	−
Colchicine	−	−	−	−8.4 ^a^

^a^ The RMSD between re-docked ligands and the corresponding X-ray crystal structure coordinates was ≤2. ^b^ These values were described in a previous study [28].

## Supplementary Materials

The following are available online. Figure S1. ^1^H-NMR spectrum of compound **5** in DMSO-d_6_. Figure S2. ^13^C-NMR spectrum of compound **5** in DMSO-d_6_. Figure S3. ^1^H-NMR spectrum of compound **6** in DMSO-d_6_. Figure S4. ^13^C-NMR spectrum of compound **6** in DMSO-d_6_. Figure S5. ^1^H-NMR spectrum of compound **10** in in CDCl_3_. Figure S6. ^13^C-NMR spectrum of compound **10** in in CDCl_3_. Figure S7. ^1^H-NMR spectrum of compound **11** in DMSO-d_6_. Figure S8. ^13^C-NMR spectrum of compound **11** in DMSO-d_6_. Figure S9. ^1^H-NMR spectrum of compound **12** in in CDCl_3_. Figure S10. ^13^C-NMR spectrum of compound **12** in in CDCl_3_.

## References

[1] Siegel R.L., Miller K.D., Jemal A. (2020). Cancer statistics, 2020. CA. Cancer J. Clin..

[2] Guo Z. (2017). The modification of natural products for medical use. Acta Pharm. Sin. B.

[3] Sutherland R.L., Watts C.K.W., Musgrove E.A. (1995). Cell cycle control by steroid hormones in breast cancer: Implications for endocrine resistance. Endocr. Relat. Cancer.

[4] Rodriguez J., Nunez L., Peixinho S., Jiménez C. (1997). Isolation and Synthesis of the First Natural 6-Hydroximino 4-en-3-one- Steroids from the Sponges. Tetrahedron Lett..

[5] Deive N., Rodriguez J., Jiménez C. (2001). Synthesis of Cytotoxic 6*E*-Hydroximino-4-ene Steroids: Structure/Activity Studies. J. Med. Chem..

[6] Ãbele E., Lukevics E. (2000). Recent Advances in the Chemistry of Oximes. Org. Prep. Proced. Int..

[7] Canário C., Silvestre S., Falcão A., Alves G. (2018). Steroidal Oximes: Useful Compounds with Antitumor Activities. Curr. Med. Chem..

[8] Hejaz H.A.M., Purohit A., Mahon M.F., Reed M.J., Potter B.V.L. (1999). Synthesis and Biological Activity of the Superestrogen (*E*)-17-Oximino-3-O-sulfamoyl-1,3,5(10)-estratriene: X-ray Crystal Structure of (*E*)-17-Oximino-3-hydroxy-1,3,5(10)-estratriene. J. Med. Chem..

[9] Allan G.M., Lawrence H.R., Cornet J., Bubert C., Fischer D.S., Vicker N., Smith A., Tutill H.J., Purohit A., Day J.M. (2006). Modification of Estrone at the 6, 16 and 17 Positions: Novel Potent Inhibitors of 17β-Hydroxysteroid Dehydrogenase Type 1. J. Med. Chem..

[10] Hartmann R.W., Hector M., Haidar S., Ehmer P.B., Reichert W., Jose J. (2000). Synthesis and Evaluation of Novel Steroidal Oxime Inhibitors of P450 17 (17alfa-Hydroxylase/C17-20-Lyase) and 5alfa-Reductase Types 1 and 2. J. Med. Chem..

[11] Pokhrel M., Ma E. (2011). Synthesis and screening of aromatase inhibitory activity of substituted C19 steroidal 17-oxime analogs. Molecules.

[12] Fischer D.S., Allan G.M., Bubert C., Vicker N., Smith A., Tutill H.J., Purohit A., Wood L., Packham G., Mahon M.F. (2005). *E*-Ring Modified Steroids as Novel Potent Inhibitors of 17β-Hydroxysteroid Dehydrogenase Type 1. J. Med. Chem..

[13] Leese M.P., Leblond B., Newman S.P., Purohit A., Reed M.J., Potter B.V.L. (2005). Anti-cancer activities of novel D-ring modified 2-substituted estrogen-3-*O*-sulfamates. J. Steroid Biochem. Mol. Biol..

[14] Payne A.H., Hales D.B. (2004). Overview of steroidogenic enzymes in the pathway from cholesterol to active steroid hormones. Endocr. Rev..

[15] Berényi Á., Minorics R., Iványi Z., Ocsovszki I., Ducza E., Thole H., Mernyák E., Frank É., Schneider G., Zupkó I. (2013). Synthesis and investigation of the anticancer effects of estrone-16-oxime ethers in vitro. Steroids.

[16] Wendlandt A.E., Yelton S.M., Lou D., Watt D.S., Noonan D.J. (2010). Synthesis and functional analysis of novel bivalent estrogens. Steroids.

[17] Cushman M., He H.-M., Katzenellenbogen J.A., Lin C.M., Hamel E. (1995). Synthesis, antitubulin and antimitotic activity, and cytotoxicity of analogs of 2-methoxyestradiol, an endogenous mammalian metabolite of estradiol that inhibits tubulin polymerization by binding to the colchicine binding site. J. Med. Chem..

[18] Lao K., Wang Y., Chen M., Zhang J., You Q. (2017). Design, synthesis and biological evaluation of novel 2-methoxyestradiol analogs as dual selective estrogen receptor modulators (SERMs) and antiangiogenic agents. Eur. J. Med. Chem..

[19] Haidar S., Klein C.D., Hartmann R.W., Chemie M., Saarlandes U., Box P.O., Saarbrücken D. (2001). Synthesis and Evaluation of Steroidal Hydroxamic Acids as Inhibitors of P450 17 (17alfa-Hydroxylase/C17-20-Lyase). Arch. Pharm. Pharm. Med. Chem..

[20] Kim S., Ma E. (2009). Synthesis of Pregnane Derivatives, Their Cytotoxicity on LNCap and PC-3 Cells, and Screening on 5α-Reductase Inhibitory Activity. Molecules.

[21] Kim S., Kim Y., Ma E. (2012). Synthesis and 5α-reductase inhibitory activity of C₂₁ steroids having 1,4-diene or 4,6-diene 20-ones and 4-azasteroid 20-oximes. Molecules.

[22] Choudhary M.I., Alam M.S., Yousuf S., Wu Y.-C., Lin A.-S., Shaheen F. (2011). Pregnenolone derivatives as potential anticancer agents. Steroids.

[23] Holland H.L., Kumaresan S., Tan L., Njar V.C.O. (1992). Synthesis of 6-Hydroximino-3-oxo Steroids, a New Class of Aromatase Inhibitor. J. Chem. Soc Perkin Trans 1.

[24] Cushman M., He H.M., Katzenellenbogen J.A., Varma R.K., Hamel E., Lin C.M., Ram S., Sachdeva Y.P. (1997). Synthesis of analogs of 2-methoxyestradiol with enhanced inhibitory effects on tubulin polymerization and cancer cell growth. J. Med. Chem..

[25] Rzheznikov V.M., Golubovskaya L.E., Minailova O.N., Osetrova I.P., Smirnova Z.S. (2003). Steroidal Nitrates: Synthesis and Antitumor Activity of of 9α, 11β-Dihydroxyestra-1,3,5(10)-triene 11-nitrates. Pharm. Chem. J..

[26] Sánchez-Sánchez L., Hernández-Linares M.G., Escobar M.L., López-Muñoz H., Zenteno E., Fernández-Herrera M.A., Guerrero-Luna G., Carrasco-Carballo A., Sandoval-Ramírez J. (2016). Antiproleferative, Cytotoxic and Apoptotic Activity of Steroidal Oximes in Cervicouterine Cell Lines. Molecules.

[27] Tang J.J., Li G., Gao J.M. (2019). Synthesis and cytotoxicity of novel steroidal C-20 oxime ester derivatives from 16-DPA. Arab. J. Chem..

[28] Canário C., Matias M., de Brito V., Santos A.O., Falcão A., Silvestre S., Alves G. (2020). Δ^9,11^-Estrone derivatives as potential antiproliferative agents: Synthesis, in vitro biological evaluation and docking studies. Comptes Rendus Chim..

[29] Stéphan E., Zen R., Authier L., Jaouen G. (1995). Improved synthesis of a protected 11-oxoestrone. Steroids.

[30] Stubenrauch G., Knuppen R. (1976). Convenient large scale preparation of catechol estrogens. Steroids.

[31] Saikia L., Baruah J., Thakur A. (2011). A rapid, convenient, solventless green approach for the synthesis of oximes using grindstone chemistry. Org. Med. Chem. Lett..

[32] Kim B.R., Sung G.H., Kim J.J., Yoon Y.J. (2013). A development of rapid, practical and selective process for preparation of *z*-oximes. J. Korean Chem. Soc..

[33] Jadhav A.D., Gade E.H., Angarkhe B.L., Durrani A. (2018). An Efficient One Pot Synthesis of Oxime by Classical Method. Int. J. Chem. Phys. Sci..

[34] Tsuchiya Y., Nakajima M., Yokoi T. (2005). Cytochrome P450-mediated metabolism of estrogens and its regulation in human. Cancer Lett..

[35] Ayan D., Maltais R., Roy J., Poirier D. (2012). A new nonestrogenic steroidal inhibitor of 17β-hydroxysteroid dehydrogenase type I blocks the estrogen-dependent breast cancer tumor growth induced by estrone. Mol. Cancer Ther..

[36] Cortés-Benítez F., Roy J., Maltais R., Poirier D. (2017). Impact of androstane A- and D-ring inversion on 17β-hydroxysteroid dehydrogenase type 3 inhibitory activity, androgenic effect and metabolic stability. Bioorganic Med. Chem..

[37] Palomino E., Heef M.J., Horwitz J.P., Polin L., Brooks S.C. (1994). Skeletal conformations and receptor binding of some 9,11-modified estradiols. J. Steroid Biochem. Mol. Biol..

[38] Andruska N., Mao C., Cherian M., Zhang C., Shapiro D.J. (2012). Evaluation of a Luciferase-based Reporter Assay as a Screen for Inhibitors of Estrogen-ERα-induced Proliferation of Breast Cancer Cells. J Biomol Screen.

[39] Robert S. (2002). DiPaola To Arrest or Not To G_2_-M Cell-Cycle Arrest. Clin. Cancer Res..

[40] Latif A.D., Gonda T., Vágvölgyi M., Kúsz N., Kulmány Á., Ocsovszki I., Zomborszki Z.P., Zupkó I., Hunyadi A. (2019). Synthesis and in vitro antitumor activity of naringenin oxime and oxime ether derivatives. Int. J. Mol. Sci..

[41] Mernyák E., Kovács I., Minorics R., Sere P., Czégány D., Sinka I., Wolfling J., Schneider G., Újfaludi Z., Boros I. (2015). Synthesis of trans-16-triazolyl-13 α-methyl-17-estradiol diastereomers and the effects of structural modifications on their in vitro antiproliferative activities. J. Steroid Biochem. Mol. Biol..

[42] Demirci S., Hayal T.B., Kıratlı B., Şişli H.B., Demirci S., Şahin F., Doğan A. (2019). Design and synthesis of phenylpiperazine derivatives as potent anticancer agents for prostate cancer. Chem. Biol. Drug Des..

[43] Koff J.L., Ramachandiran S., Bernal-Mizrachi L. (2015). A time to kill: Targeting apoptosis in cancer. Int. J. Mol. Sci..

[44] McLaughlin F., Finn P., La Thangue N.B. (2003). The cell cycle, chromatin and cancer: Mechanism-based therapeutics come of age. Drug Discov. Today.

[45] Toné S., Sugimoto K., Tanda K., Suda T., Uehira K., Kanouchi H., Samejima K., Minatogawa Y., Earnshaw W.C. (2007). Three distinct stages of apoptotic nuclear condensation revealed by time-lapse imaging, biochemical and electron microscopy analysis of cell-free apoptosis. Exp. Cell Res..

[46] Parker A.L., Kavallaris M., McCarroll J.A. (2014). Microtubules and their role in cellular stress in cancer. Front. Oncol..

[47] Yang C.P.H., Horwitz S.B. (2017). Taxol^®^: The first microtubule stabilizing agent. Int. J. Mol. Sci..

[48] Cabral F., Billstrom A., Hartley-Asp B. (1993). Estramustine Depolymerizes Microtubules by Binding to Tubulin. Cancer Res..

[49] Lima W.E.A., Pereira A.F., Castro A.A., Cunha E.F.F., Ramalho T.C. (2016). Flexibility in the Molecular Design of Acetylcholinesterase Reactivators: Probing Representative Conformations by Chemometric Techniques and Docking/QM Calculations. Lett. Drug Des. Discov..

[50] Giacoppo J.O.S., França T.C.C., Kuča K., da Cunha E.F.F., Abagyan R., Mancini D.T., Ramalho T.C. (2015). Molecular modeling and in vitro reactivation study between the oxime BI-6 and acetylcholinesterase inhibited by different nerve agents. J. Biomol. Struct. Dyn..

[51] de Paula R.L., de Almeida J.S.F.D., Cavalcante S.F.A., Gonçalves A.S., Simas A.B.C., Franca T.C.C., Valis M., Kuca K., Nepovimova E., Granjeiro J.M. (2018). Molecular modeling and in vitro studies of a neutral oxime as a potential reactivator for acetylcholinesterase inhibited by paraoxon. Molecules.

[52] Day J.M., Tutill H.J., Purohit A., Reed M.J. (2008). Design and validation of specific inhibitors of 17β-hydroxysteroid dehydrogenases for therapeutic application in breast and prostate cancer, and in endometriosis. Endocr. Relat. Cancer.

[53] Dorléans A., Gigant B., Ravelli R.B.G., Mailliet P., Mikol V., Knossow M. (2009). Variations in the colchicine-binding domain provide insight into the structural switch of tubulin. Proc. Natl. Acad. Sci. USA.

[54] Bueno O., Gallego J.E., Martins S., Andrea E.P., Gago F., Gómez-sanjuan A., Camarasa M., Barasoain I., Steinmetz M.O., Díaz J.F. (2018). High-affinity ligands of the colchicine domain in tubulin based on a structure-guided design. Sci. Rep..

[55] Kumar B.S., Raghuvanshi D.S., Hasanain M., Alam S., Sarkar J., Mitra K., Khan F., Negi A.S. (2016). Recent Advances in chemistry and pharmacology of 2-methoxyestradiol: An anticancer investigational drug. Steroids.

[56] Santaniello E., Ravasi M., Ferraboschi P. (1983). A-Ring nitration of estrone. J. Org. Chem..

[57] Bose A., Sanjoto W.P., Villarreal S., Aguilar H., Banik B.K. (2007). Novel nitration of estrone by metal nitrates. Tetrahedron Lett..

[58] Tanenbaum D.M., Wang Y., Williams S.P., Sigler P.B. (1998). Crystallographic comparison of the estrogen and progesterone receptor’s ligand binding domains. Proc. Natl. Acad. Sci. USA.

[59] Hernandez-Guzman F.G., Higashiyama T., Pangborn W., Osawa Y., Ghosh D. (2003). Structure of human estrone sulfatase suggests functional roles of membrane association. J. Biol. Chem..

[60] Aka J.A., Mazumdar M., Chen C.-Q., Poirier D., Lin S.-X. (2010). 17β-Hydroxysteroid Dehydrogenase Type 1 Stimulates Breast Cancer By Dihydrotestosterone Inactivation in Addition To Estradiol Production. Mol. Endocrinol..

[61] Ravelli R.B.G., Gigant B., Curmi P.A., Jourdain I., Lachkar S., Sobel A., Knossow M. (2004). Insight into tubulin regulation from a complex with colchicine and a stathmin-like domain. Nature.

[62] Meng X.-Y., Zhang H.-X., Mezei M., Cui M. (2011). Molecular Docking: A Powerful Approach for Structure-Based Drug Discovery. Curr. Comput. Aided-Drug Des..

[63] Carugo O. (2003). How root-mean-square distance (r.m.s.d.) values depend on the resolution of protein structures that are compared. J. Appl. Crystallogr..

