# Supplementary material for: New Estrone Oxime Derivatives: Synthesis, Cytotoxic Evaluation and Docking Studies
Source: Molecules. 2021 May 4;26(9):2687. doi: 10.3390/molecules26092687 (PMC8125528; doi:10.3390/molecules26092687)
Supplement: Supplementary file 1 [file molecules-26-02687-s001.zip › molecules-1178425-supplementary.pdf]

Supplementary Material

New estrone oxime derivatives: synthesis, cytotoxic evaluation and docking studies

Catarina Canário [1], Mariana Matias [1], Vanessa de Brito [1], Adriana O. Santos [1], Amílcar Falcão [2,3], Samuel Silvestre [1,4,*] and Gilberto Alves [1]

[1] CICS-UBI – Health Sciences Research Centre, University of Beira Interior, Covilhã, Portugal;
[2] Laboratory of Pharmacology, Faculty of Pharmacy, University of Coimbra, Coimbra, Portugal;
[3] CIBIT - Coimbra Institute for Biomedical Imaging and Translational Research, University of Coimbra, Coimbra, Portugal;
[4] CNC – Center for Neuroscience and Cell Biology, University of Coimbra, Coimbra, Portugal.
* Correspondence: sms@ubi.pt

NMR Data of Compounds

1. Spectra of compound 5

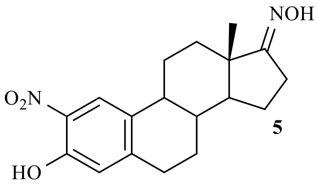

5

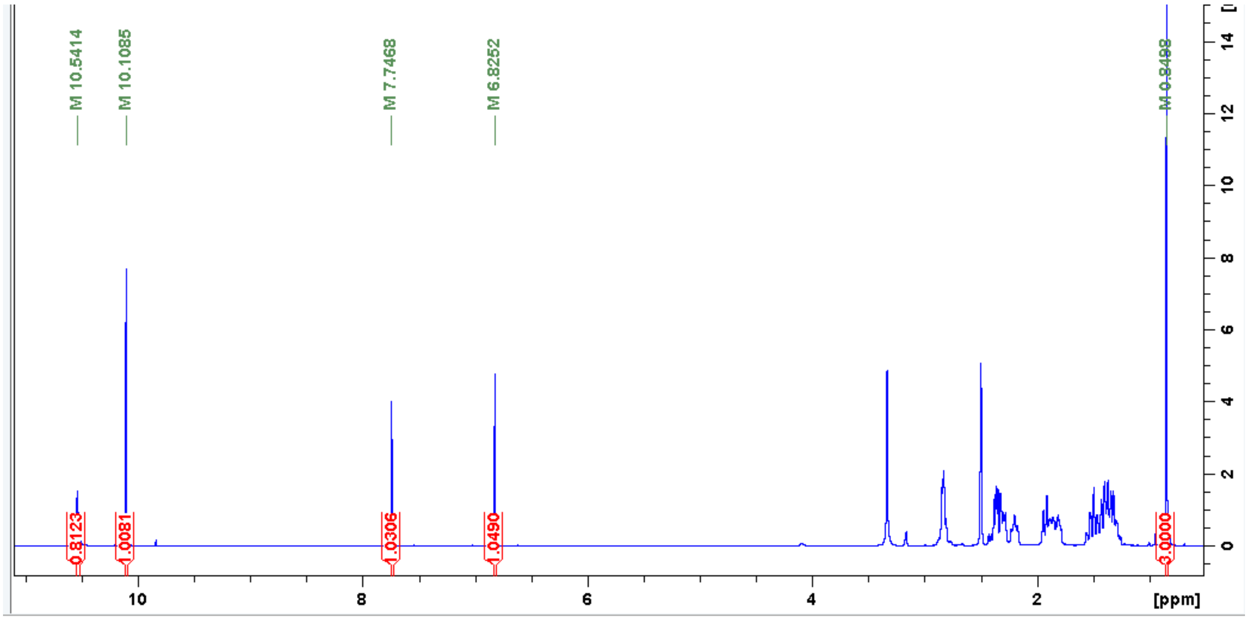

Figure S1. ^{1}H-NMR spectrum of compound **5** in DMSO-d_6.

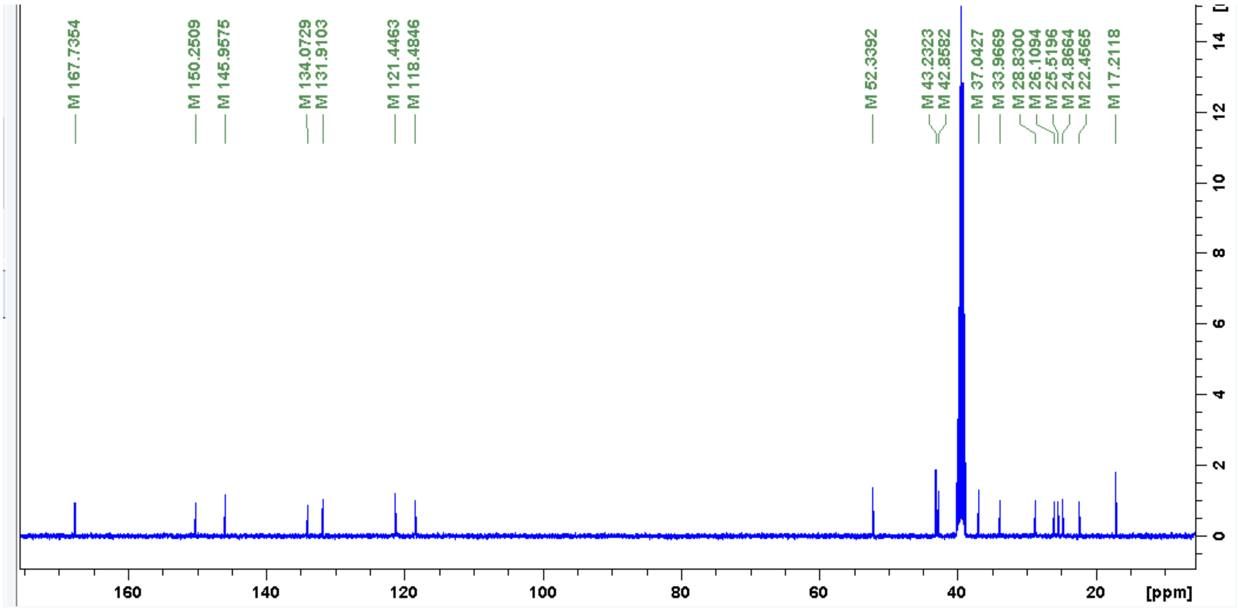

Figure S2. ^{13}C-NMR spectrum of compound **5** in DMSO-d6.

2. Spectra of compound 6

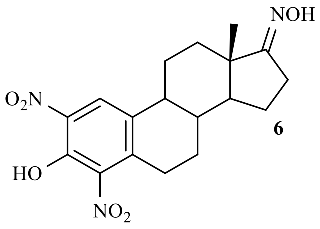

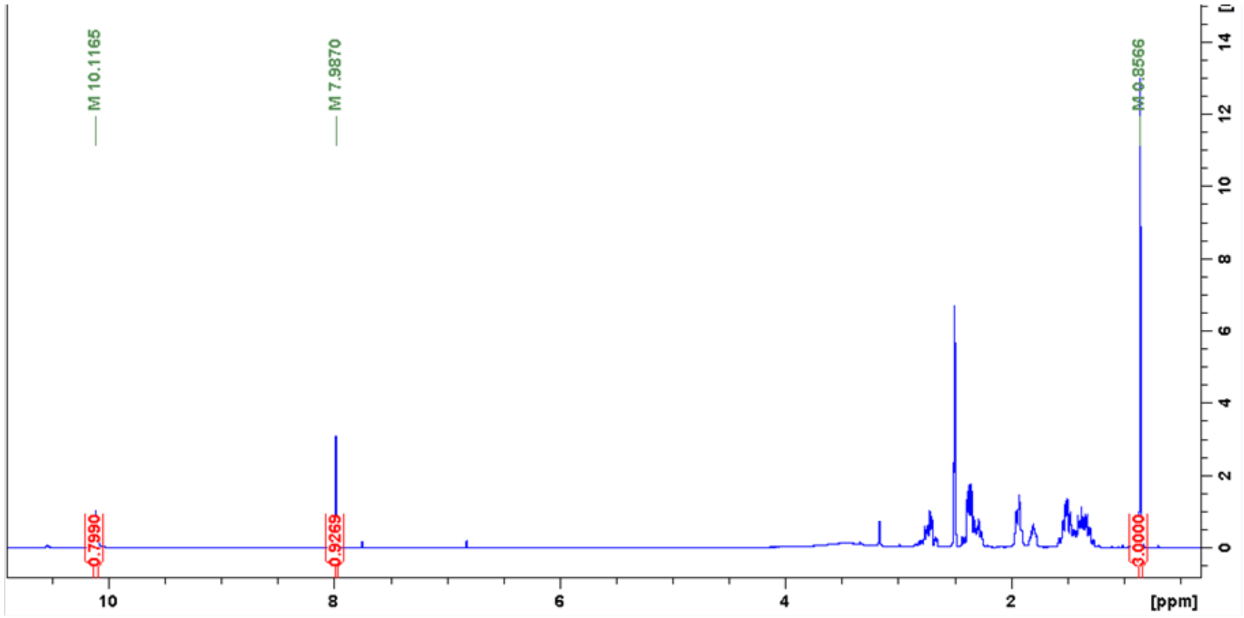

Figure S3. ^{1}H-NMR spectrum of compound **6** in DMSO-d_6.

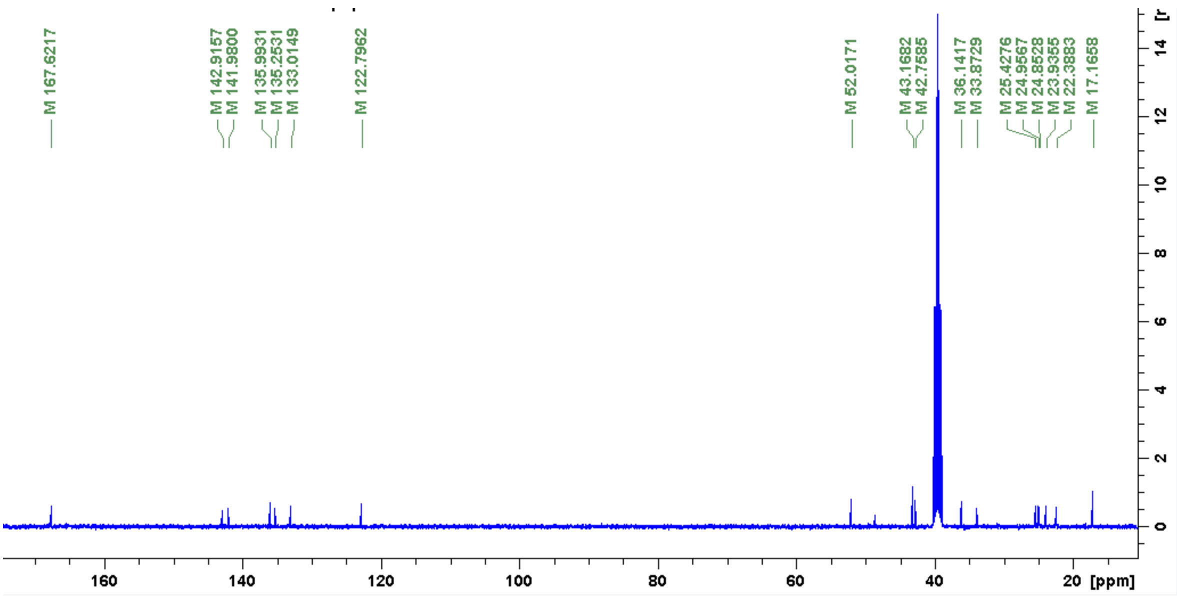

Figure S4. ^{13}C-NMR spectrum of compound **6** in DMSO-d_6.

3. Spectra of compound 10

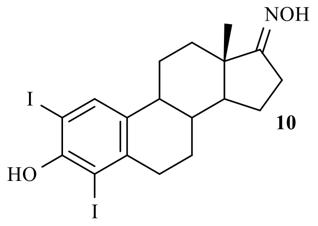

10

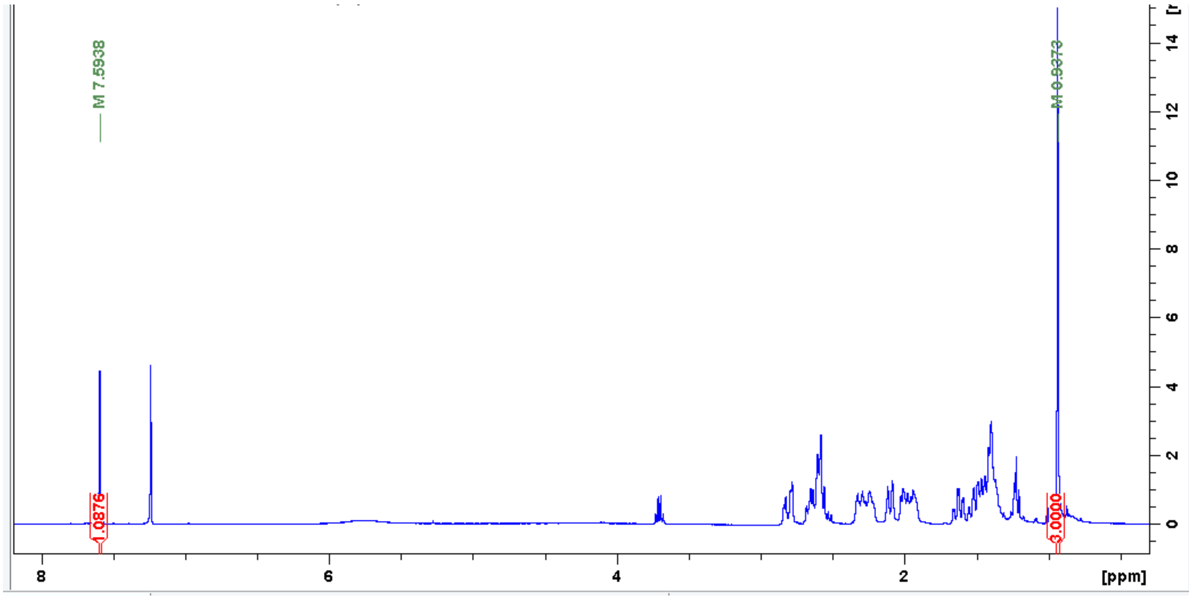

Figure S5. ^{1}H-NMR spectrum of compound **10** in in $CDCl_3$.

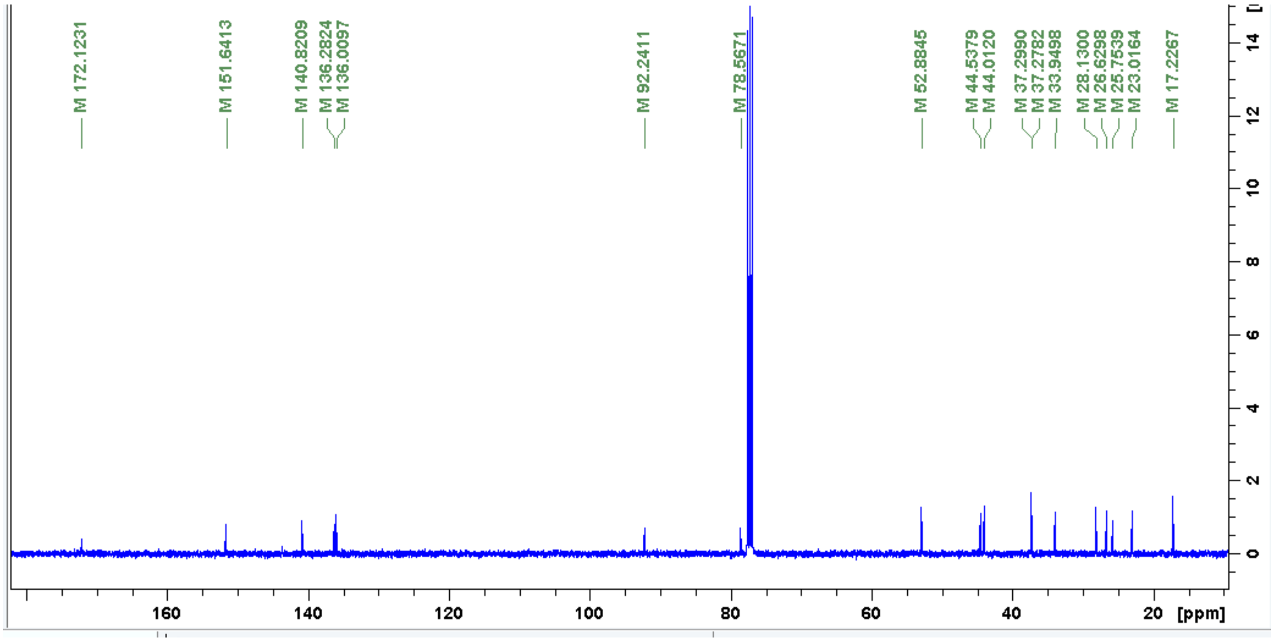

Figure S6. ^{13}C-NMR spectrum of compound **10** in in $CDCl_3$.

4. Spectra of compound 11

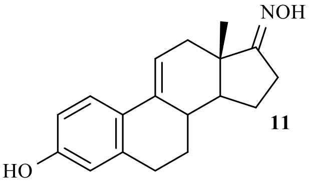

11

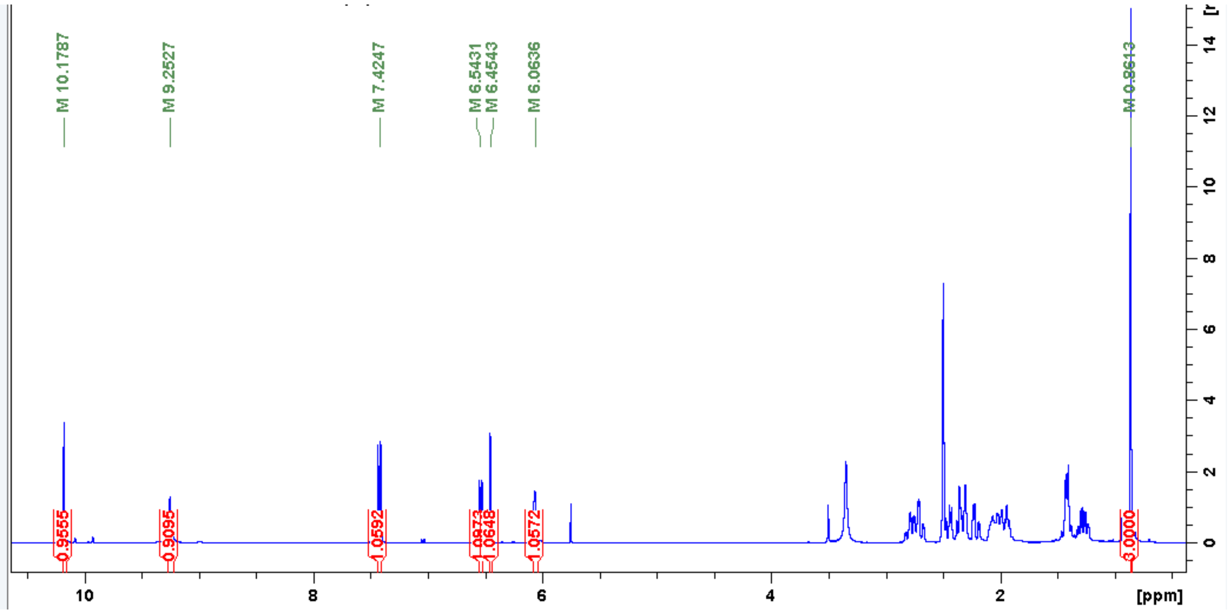

Figure S7. ^{1}H-NMR spectrum of compound **11** in DMSO-d6.

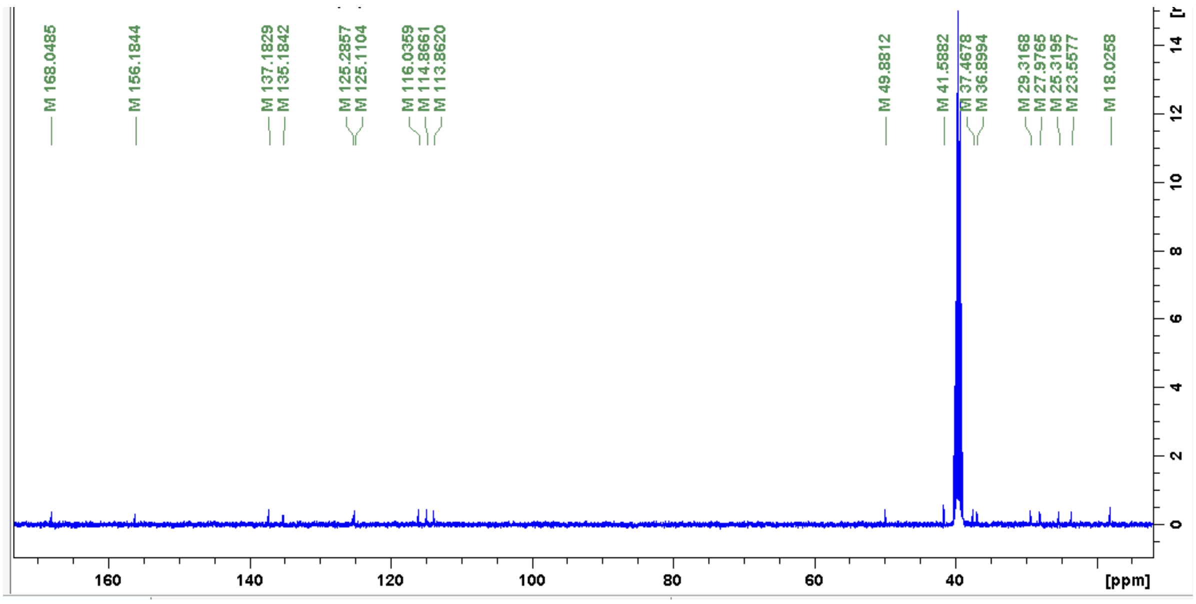

Figure S8. ^{13}C-NMR spectrum of compound **11** in DMSO-d_6.

5. Spectra of compound 12

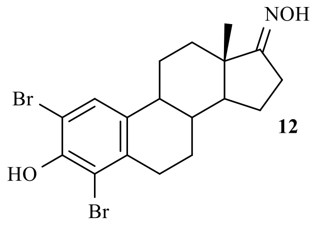

12

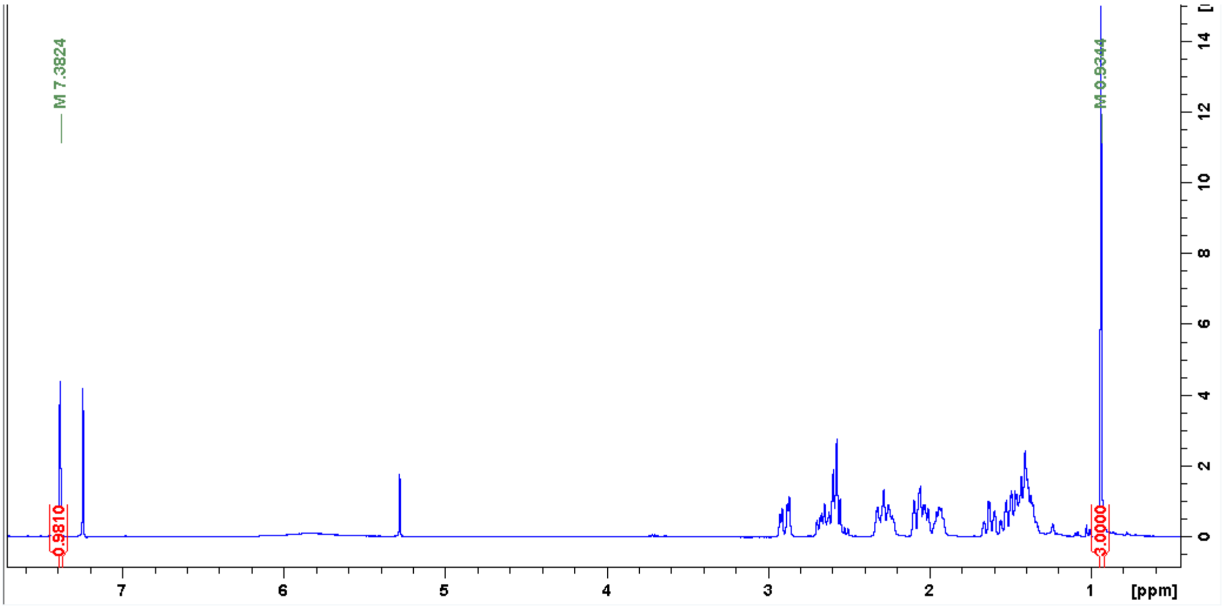

Figure S9. ^{1}H-NMR spectrum of compound **12** in in $CDCl_3$.

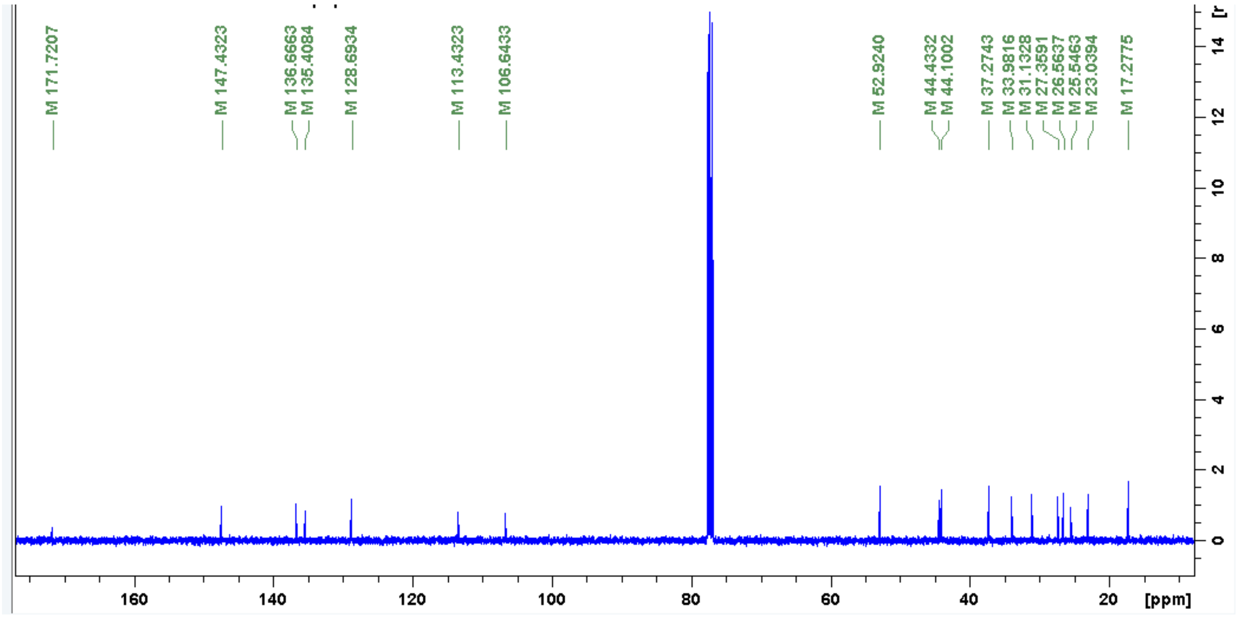

Figure S10. ^{13}C-NMR spectrum of compound **12** in in $CDCl_3$.
